# A standardised classification scheme for the Mid-Holocene Toalean artefacts of South Sulawesi, Indonesia

**DOI:** 10.1371/journal.pone.0251138

**Published:** 2021-05-26

**Authors:** Yinika L. Perston, Mark Moore, Suryatman, Michelle Langley, Budianto Hakim, Adhi Agus Oktaviana, Adam Brumm

**Affiliations:** 1 Australian Research Centre for Human Evolution, School of Environment and Science, Griffith University, Brisbane, Queensland, Australia; 2 Stone Tools and Cognition Hub, Archaeology and Palaeoanthropology, University of New England, Armidale, Australia; 3 Balai Arkeologi Sulawesi Selatan, Makassar, Sulawesi, Indonesia; 4 Forensics and Archaeology, School of Environment and Science, Griffith University, Brisbane, Queensland, Australia; 5 Place Evolution and Rock Art Heritage Unit, Griffith Centre for Social and Cultural Research, Griffith University, Gold Coast, Australia; 6 Pusat Penelitian Arkeologi Nasional, Jakarta, Java, Indonesia; James Cook University - Cairns Campus, AUSTRALIA

## Abstract

The archaeology of Sulawesi is important for developing an understanding of human dispersal and occupation of central Island Southeast Asia. Through over a century of archaeological work, multiple human populations in the southwestern region of Sulawesi have been identified, the most well-documented being that of the Mid- to Late Holocene ‘Toalean’ technological period. Archaeological models for this period describe a population with a strong cultural identity, subdivided into groups living on the coastal plains around Maros as well as dispersed upland forest dwellers, hunting endemic wildlife with bow-and-arrow technology. It has been proposed that the Toaleans were capable of vast water-crossings, with possible cultural exchange with northern Australia, Java, and Japan. This Toalean paradigm is built almost exclusively on existing interpretations of distinctive Toalean stone and bone artefact technologies, constructed on out-dated 19^th^ and 20^th^ century theory. Moreover, current definitions of Toalean artefact types are inconsistently applied and unsystematic, and the manufacturing sequence has historically been poorly understood. To address these problems in existing artefact models and typologies, we present a clarified typology of the Toalean artefacts of South Sulawesi, and describe the technical aspects of artefact production. This typology provides a tool for standardising research and will facilitate more meaningful assessments of material culture repertoires and more reliable assessment of spatial and temporal changes for the region.

## Introduction

The southwestern peninsula of Sulawesi, or ‘South Sulawesi’, has frequently been cited as a potential key point for cultural connections during the Mid-Holocene. Archaeological examples of small backed microliths, osseous points, and hollow-based stone points from the peninsula have been likened to the assemblages of Japan, Java, and Australia [e.g. 1, 2–4]. Based on these observations, models have been developed suggesting pre-‘Austronesian’ groups dispersed from Japan or mainland Asia, through Sulawesi, to central Java, as well as to Australia where they introduced dingos (*Canis lupus dingo*) and Pama-Nyungan languages [1, 4–7]. These models often draw on perceived technological similarities between the stone and bone artefacts across these disparate regions, raising two questions: 1) are the technologies truly similar, and 2) if so, how do we explain these similarities? This paper focuses on the first of these questions, and works to establish a framework for a more objective understanding of the second.

While lithic technologies are central to current models of human occupation of Mid-Holocene South Sulawesi, known as the ‘Toalean’ period, analyses of the stone artefacts are often composed of preliminary descriptions that largely rely on reference to a small number of distinctive artefact forms or types. There are a few exceptions [8–11], although these are limited in scope. A problem arises when generalisations are built on these studies, however, as many of these artefact types have not yet been clearly or consistently defined, a common issue for lithic studies [12]. Definitions and nomenclature for the characteristic Toalean tools–Maros points, backed microliths, bone points, and even cores–differ dramatically between reports, making inter-site comparisons problematic. To address this problem, this paper provides a systematic reappraisal of the existing terminology applied to the artefacts of South Sulawesi’s Toalean material assemblages. The reappraisal includes detailed descriptions of the technological processes behind producing each artefact class, as reconstructed by integrating data and observations collected over five years of fieldwork with the existing literature that has been accumulating for a little over a century. A newly-recognised artefact form, the ‘sawlette’, is also described. A descriptive approach is employed to produce morphological categories for systematically reappraising the archaeological models for this period and for addressing broader questions about the region’s role in narratives of Mid-Holocene human dispersals through Wallacea.

A typological approach focussed on artefact classes (‘types’) dominates lithic studies in Indonesia and continues to be the standard approach throughout the region. Critiques of the typological approach focus on assumptions in the earlier literature about how types must reflect mental categories, although it is possible to use typologies without these assumptions [13]. Materialist classifications emphasise variation within a population, but the attributes that underpin materialist approaches are identified using typological methods. For instance, the materialist approach to studying microlith symmetry by Hiscock [14] is based on the typological division of microliths from other types of retouched flakes, but without adopting mentalist assumptions about microlith design or use. Further, Hiscock’s [14] materialist approach to analysing whole assemblages divides artefacts into general ‘classifications’ by applying standard typological approaches based on pattern-matching to identify recurring attributes; this is explicitly accomplished without adopting mentalism. As is broadly true of scientific disciplines that attempt to describe variation, the formation of descriptive units is unavoidable in lithic studies [15]. In this paper, we attempt to refine and improve the nominal descriptive units imposed on stone artefact assemblages from southwestern Sulawesi, but, following Hiscock [14], we do so without adopting a mentalist position. Our goal is to provide an improved context for describing the archaeological record of the region, thus providing a more robust foundation for addressing what this variation might mean chronologically and behaviourally.

## Background

The Mid- to Late- Holocene period of human occupation in South Sulawesi is known as the ‘Toalean’ [16], ‘Toalian’ [17, 18], or *Toala* (Indonesian), or occasionally the region’s ‘Mesolithic’ period [e.g. 16, 19, 20]. Known Toalean sites are largely concentrated in the caves of the limestone karst system that runs through the lowland plains of the *Maros* and *Pangkajene dan Kepulauan* (or ‘Pangkep’) regencies, immediately north-east of Makassar, but they also occur in the adjoining administrative regencies. The name ‘Toalean’ originates from the Bugis words *tau alek* [21, 22], or ‘people of the forest’, and reflects an initial assumption that the archaeological deposits were generated by the recent ancestors of a group of people encountered in forest caves in the south Bone highlands during the early 1900s [23], although later work revealed the antiquity of the deposits and thus this link seems unlikely [20, 24]. Arguably the term ‘Toalean’ should be used in a narrow sense, to describe a material culture tradition [10] or technocomplex [25]; however, it is commonly used broadly to describe both the Mid-Holocene cultural group of South Sulawesi and their technologies, which date from around 8 thousand years (ka) ago to approximately 1.5 ka ago [20].

Current understandings of the Toalean period are based on a large body of excavations, and South Sulawesi is one of the most heavily excavated regions in Indonesia. Archaeological research in South Sulawesi began at the turn of the 20^th^ century with the work of the Sarasin cousins [23], and since then dozens of cave sites and open sites with Toalean deposits or surface finds have been identified and published by Indonesian and international research groups [26, 27]. Many more sites have been excavated or surveyed but not yet published, with reports and databases lodged in government organisations and universities in Indonesia and overseas. Sites are classified as Toalean based on the stone and bone artefacts they contain, as the assemblages are technologically distinct from other assemblages.

Toalean assemblages include several diagnostic artefact types that distinguish them from earlier or later deposits. These distinctive Toalean artefacts comprise of hollow-based lithic ‘Maros points’ with denticulated edges, small osseous or ‘bone’ points, and backed microliths. Early studies also report on tanged and ‘pedunculated’ (stemmed) blades [van Stein Callenfels in 2 p. 113], although these have since been identified as broken flakes [28]. Toalean artefacts are often associated with skeletal remains of Sulawesi warty pigs (*Sus celebensis*) [29, 30], one of two still-extant species of suid that are endemic to the island (the other is the babirusa; *Babyrousa celebensis* [31]). Toalean art does not appear to be prolific, and portable examples appear to be limited to isolated examples including an engraved osseous point from Ulu Leang 1 [28] and a painted shell at Leang Rakkoe [32], and no Toalean cave art has been identified.

This profile contrasts with Late Pleistocene deposits, which lack the diagnostic Toalean artefact types and are instead dominated by unmodified flakes, small bipolar artefacts, and large cores [33–36]. The limestone caves of Maros and Pangkep are rich in parietal ochre paintings, and while these were initially assumed to be Toalean [16, 37], recent dates obtained from multiple paintings have all returned Pleistocene origins [38–41]. Similarly, archaeological excavations at the Late Pleistocene site of Leang Bulu Bettue have revealed several examples of portable ‘art’ [34, 42, 43].

Post-Toalean or ‘Neolithic’ changes in archaeological assemblages are seen to represent the arrival of Austronesian-speaking or ‘Nusantoa’ migrants [see 44 for a discussion on terminology]. Assemblages from this period include ground stone axes and pottery [45], and may be associated with black line cave art including *kangkang* [Basran, pers. comm.] style anthropomorphic stick figures with splayed, angular limbs [34]. Neolithic deposits are often mixed with Toalean artefacts, either through disturbance [46], possible cultural diffusion, and/or the existence of trade and exchange between the indigenous foragers and immigrant farming communities [6, 47, 48].

**Fig 1:**
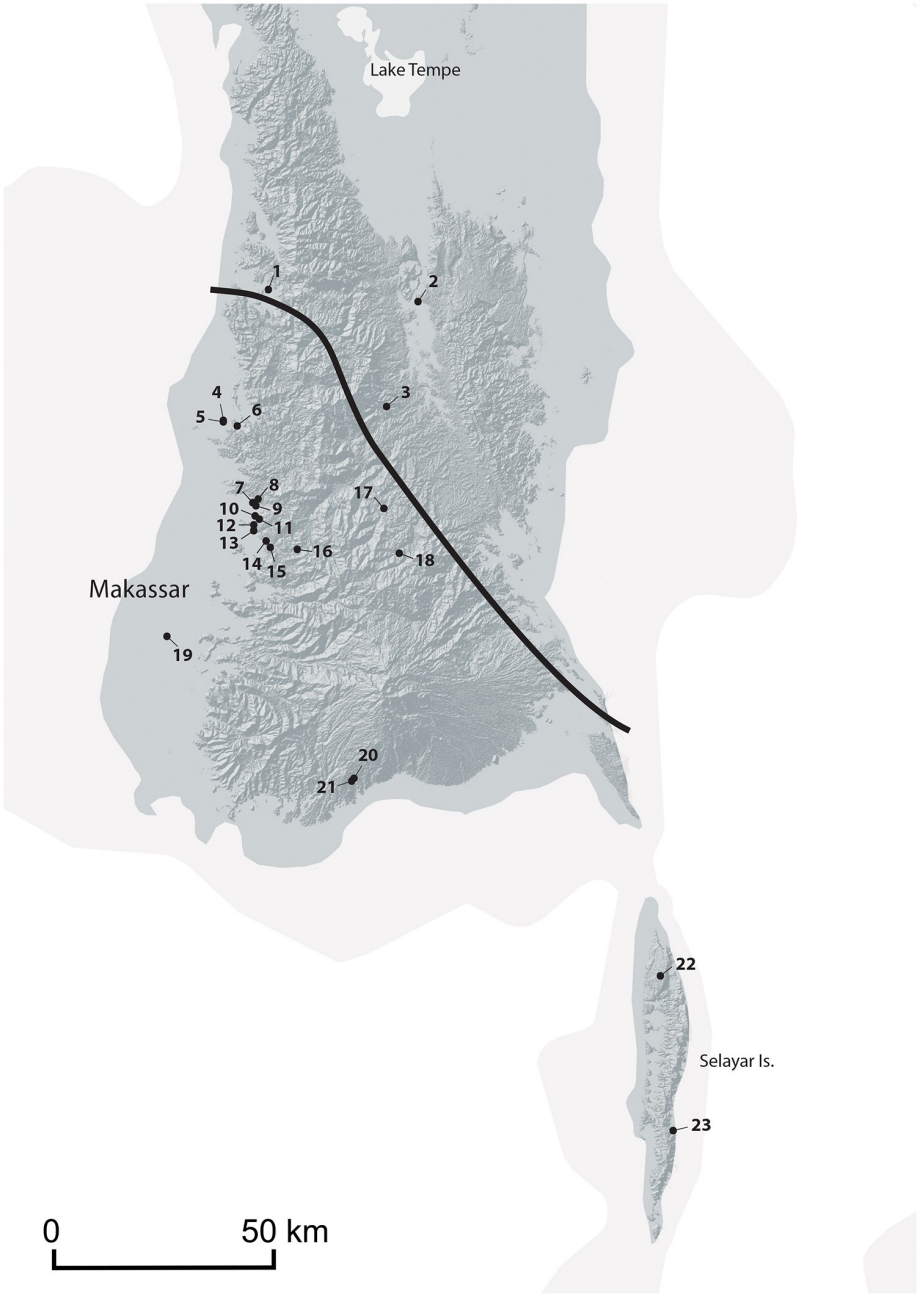
Map of South Sulawesi, Indonesia, showing sites mentioned in the text. **1**. Ralla, **2**. Mallinrung, **3**. Leang Panninge, **4**. Leang Bulu’ Sipong 4, **5**. Leang Bulu’ Sipong 1, **6**. Leang Sakapao, **7**. Leang Rakkoe, **8**. Leang Lompoa, **9**. Leang Lambatorang, **10**. Leang Pajae, **11**. Ulu Leang 1, **12**. Leang Bulu Bettue, **13**. Leang Burung 1 & 2, **14**. Leang Jarie, **15**. Leang Karassa, **16**. Tallasa, **17**. Lamoncong, **18**. Balang Metti, **19**. Pammangkulang Batua, **20**. Pangnganikang, **21**. Batu Ejayya, **22**. Silolo, **23**. Jammeng. Bulbeck’s “Classical Toalean” Line [20], shown in black, marks the known extent of hollow-based denticulated Maros points and backed microliths which he has proposed may represent a ‘southwest’ Toalean entity [e.g. 52]. Recently both artefact types have been recovered from the site on Leang Panninge, just north of this Line [54]. Basemap made with Natural Earth 2009–2020 under CC-O, DEM created from STRM files available from the USGS Earth Explorer, image compiled by Kim Newman and Yinika L Perston.

Toalean culture appears to have been confined to the southern third of the southwest peninsula of Sulawesi, as no Toalean sites have been found north of Lake Tempe [20 p. 93] (Fig 1). The southern extent may be defined by the presence of six Maros points recovered from Selayar island at the open sites of Silolo, Batangmata Sapo, and Sinagari, Bontosikuyu, and at the Jammeng rockshelter, Bontosikuyu [25, 49, pers. obs. BH]. The island lies approximately 30km from the mainland, suggesting possible open-water maritime capabilities during this period [20].

Within the Toalean range, Bulbeck identifies two different entities, which roughly correspond to the range of modern day Makasar and Bugis language groups [20, 50, 51]. According to this model, Toaleans of the ‘southwest’ were complex hunter-gatherers with high population densities living on the coastal plains on the south and southwest, and they produced the ‘classic’ Toalean artefacts: backed microliths and Maros points, as well as bone points and ‘pirri points’. *S*. *celebensis* remains dominate the faunal assemblages. In contrast, the ‘northeast’ Toaleans of the central region of South Sulawesi and the eastern coast were organised into smaller and more scattered foraging societies that produced so-called pirri points and bone points, but lacked backed microliths and Maros points, and the faunal assemblages include more forest-based species [52]. However, new research is leading to a reappraisal of this model, with emerging evidence for classic Toalean artefacts in the northeast highlands [29, 53]. Moreover, as this paper will highlight, definitions of what constitute a Maros point currently differ widely, meaning that distinctions in pirri point and Maros point distributions may be inaccurate.

Bulbeck’s model, and other like it, rely on consistent application of artefact definitions, especially the term ‘Maros point’. However, a review of the literature reveals a range of different ways in which this term is defined as some authors apply the title to almost any Toalean stone points while other advocate strict limitations on the application according to shape, presence and depth of a basal hollow, and distribution of edge denticulations [8, 50, 55]. As stone points of South Sulawesi are highly variable in shape and features, leading some authors to employ categories including ‘pirri points’ and ‘Malindrung/Mallinrung points’, though these were not universally adopted.

Some authors have worked around this issue by relying only on their own analyses; however, these can also be critiqued. During the 1980s an attempt was made at an inter-site comparison by a single analyst, Chapman [3, 8]. In this largely taxonomic study of the lithic technology at three Toalean sites, Chapman claimed to find a common Toalean technological tradition that was modified over time and between sites. Here the low number of blade-like flakes was interpreted as indicating that “skilful knapping was not at a premium” during the Toalean period [3 p. 100]. It was also remarked that a complete classification of Maros points is not possible “without some knowledge of their manufacturing sequence” [3 p. 52]. Our paper provides the requested model of Maros point manufacturing sequences, and demonstrates how Toalean stone-flaking in-fact reflects a high level of control and skill, as well as suggesting that there is no reliable evidence that deliberate blade production occurred during the Toalean.

Similarly, in the 1990s a technological assessment attempted to compare the cores, points, and backed microliths between three Toalean sites [10]. Here, cores were defined as only those artefacts with no positive percussive features, potentially heavily skewing the analysis if core reduction was done on large flake blanks imported to one of the sites from an external quarry space. More recent work this century has provided some observations on production methods, especially of backed microliths from Balang Metti [53], and here we assess and build on that work.

Finally, this paper also addresses organic tools, in particular osseous point production. These points are fairly ubiquitous in Toalean assemblages, but relatively overlooked as a result of modern material-based classification systems. Osseous points have been assessed in some detail by Olsen and Glover as part of a wider study [56], however their sample was small and focussed on experimental replications and the possible functions of the artefacts. In contrast, our work discusses the osseous point production process in greater detail and provides new data on the use of animal teeth as a raw material.

## Methodology

The typological classification system developed here is polythetic [e.g. 13], employing both morphological and technological features; that is, types are identified not only by the overall form of the artefacts but also the methods of production. This approach results in the division of certain former typological groupings, while merging others. This classification scheme draws on data in existing literature, and the overall approach is to refine, collate, and clarify categories rather than replacing them outright.

This study introduces new data and observations from the analysis of 1,739 Toalean-age lithic artefacts from excavations in the Maros and Pangkep regencies at the limestone caves and shelters of Leang Pajae (unpublished excavations 2018), Leang Rakkoe [32], Leang Panninge [29, 54], Leang Bulu’ Sipong 1 (unpublished excavations 2018), Leang Bulu Bettue (unpublished excavation 2017), Leang Jarie [57–59], and surface collections from Leang Lambatorang and Leang Lompoa caves, and the open site of Tallasa. Metric data was taken from unbroken dimensions. Data for the maximum size of retouching scars was taken by measuring the largest flake scar on each shaped piece, and a sample of one unbroken denticulation was selected for measurement from each denticulated artefact. Denticulations here refers to stone teeth separated by notches that are equal to or narrower than the width of the teeth [after 60]. Osseous points from Leang Bulu’ Sipong 1 and Leang Pajae are also included in the assessment.

Following principles proposed by Wright [61], classes are based on explicit, easily-reproduced attributes and existing terms are retained except where they are misleading or inconsistent. Names for each type are also provided in Indonesian, the most widely understood language of South Sulawesi.

Descriptive method and terminology of osseous points follows Langley [62] with identification of taphonomic and anthropogenic features following previous osseous tool studies [e.g. 63–68]. Identification of the material relies on observation of physical attributes under low magnification and comparison to faunal examples. Each of the osseous artefacts was examined using a Zeiss Stemi-508 stereomicroscope fitted with an AxioCam 105 camera. Macrophotography was undertaken using a Canon digital SLR camera, while metrics were gathered using Mitutoyo (CD-6″CX) digital callipers with the jaws covered in a layer of plastic coating to prevent damage to the artefacts.

## Results

The results of our analysis suggest that chert was the favoured raw material during the Toalean period. Among the assemblages analysed, 93% of artefacts were made on chert (*n* = 1617, 93%) [see also 8, 53, 69], also known as silicified limestone or flint in some studies [e.g. 2, 70], with the remainder being formed on limestone (3%), as well as chalcedony, volcanic stones, metasedimentary materials, and silicified wood (<4%, combined). The chert likely originated as nodules embedded in the surrounding limestone, which were then exposed in riverbeds and cave walls. Occasionally artefacts have become white and desilicified post-deposition, probably from prolonged waterlogging [3 p. 117].

We also conducted an unpublished heat-treatment experiment in 2017, using speckled brown nodular chert from the vicinity of the Ralla open site in the Barru regency [71], and observed that controlled heating improves the ease of flaking, resulting in increased internal lustre but no colour change. Many Toalean artefacts show heat damage in the form of potlids and heat crazing, which has previously been interpreted as heat treatment [10]. However, these features suggest uncontrolled burning post-production, and to date no clear evidence in the form of differential lustre has been discovered to support deliberate heat-treatment during the Toalean period.

### Non-diagnostic lithic artefacts

While the primary goal of this study is to provide a consistent typology of diagnostic Toalean artefact types, these classes only form a small percentage of most Toalean assemblages. During this study the following observations were also made on the accompanying cores, flakes, and bipolar artefacts, in order to provide a complete picture of Toalean knapped technology.

#### Cores [*batu inti*]

Although a fairly standard artefact type, the definition of a core can vary widely [72]. For example, while analysing artefacts from the Toalean sites of Pammangkulang Batua, Leang Karassa, and Leang Burung 1, Pasqua and Bulbeck [10] did not classify artefacts as cores if they displayed any positive percussion features. Flaked artefacts that displayed positive percussion features were classified as either *debitage* or retouched pieces [10], a method similarly proposed elsewhere [e.g. 12, 73]. However, while this approach is systematic and objective, it may obscure important aspects of the reduction strategies; for example, under this system, very large flake blank-based artefacts such as horsehoof cores would be grouped with small retouched flakes, backed microliths, and projectile points, and separated from all other similarly-sized flake-producing cores that may have served similar functions. Conversely, choppers made on cobbles would be classified into a different group to morphologically similar tools made on large flakes. Therefore, for the purposes of our paper, a core is defined as any artefact from which one or more flakes has been deliberately removed, regardless of the origin of the blank. By definition this would also grade into retouched flakes and pressure flaked pieces; however, the use of subclasses addresses any possible confusion.

#### Unmodified flakes [*serpih*]

Flakes in Toalean assemblages are typically small and produced by ‘least effort’ approaches to core reduction [74, 75 p. 225–226], although as we will show they were modified into a range of complex tools. The majority of flaking was done by striking a core directly with a hammerstone [9]. Flakes are fairly thin and flat, and occasionally the exterior platform edge was trimmed (overhang removal) or small flakes struck across the platform (platform preparation) before the flake was struck [8, current analysis]. Some artefacts show edge gloss, which initial studies suggest may be from processing bamboo or wood [3, 76] and/or soft stemmed plants possibly including wild grains [77]. Pasqua and Bulbeck [10] noted a lack of microscopic residue or phytolith analyses on Toalean tools and this potential remains unexplored.

#### ‘Blades’ [*bilah*]

Here we argue that blades did not form part of the Toalean reduction sequence. A blade is a parallel-sided flake measuring at least twice as long as it is wide [12, 78]. Although blades may result from specialised core reduction [e.g. 79–82], they can also be produced by chance as part of the continuum of flake to length ratios [83, 84]. While the Toalean was initially classified as a blade industry [van Stein Callenfels in 24], recent work has concluded that the lack of definite blade cores and the low proportions of blades do not suggest a Toalean blade industry [8–10, 20, 85]. Furthermore, a study of the distribution of length to width ratios of flakes recovered from Leang Jarie empirically demonstrates that blades are part of a continuum of flake shapes [57]. Current evidence therefore does not suggest any deliberate blade production industry during in South Sulawesi during the Mid-Holocene.

#### ‘Scrapers’ [*penyerut*]

Some flake artefacts from Toalean assemblages have varying degrees of retouch, and the more regular of these are commonly described as scrapers; however this approach may be problematic. Several authors have reported the occurrence of lithic ‘scrapers’ in Toalean assemblages [e.g. 7, 8, 28, 69]. Scrapers have regular, unifacial retouch along one or more margin [e.g. 86 p. 167], which can be difficult to define when this grades into other retouched artefacts, cores, and utilised pieces. The term itself is also problematic, as it implies that the artefacts were used in scraping activities however they may have served a range of functions [87]. As yet there have only been limited functional studies of Toalean assemblages [77]. Nor are ‘scrapers’ unique to the Toalean period, being reported from Pleistocene deposits as well as beyond the known Toalean geographical distribution [e.g. 33, 88].

**Fig 2:**
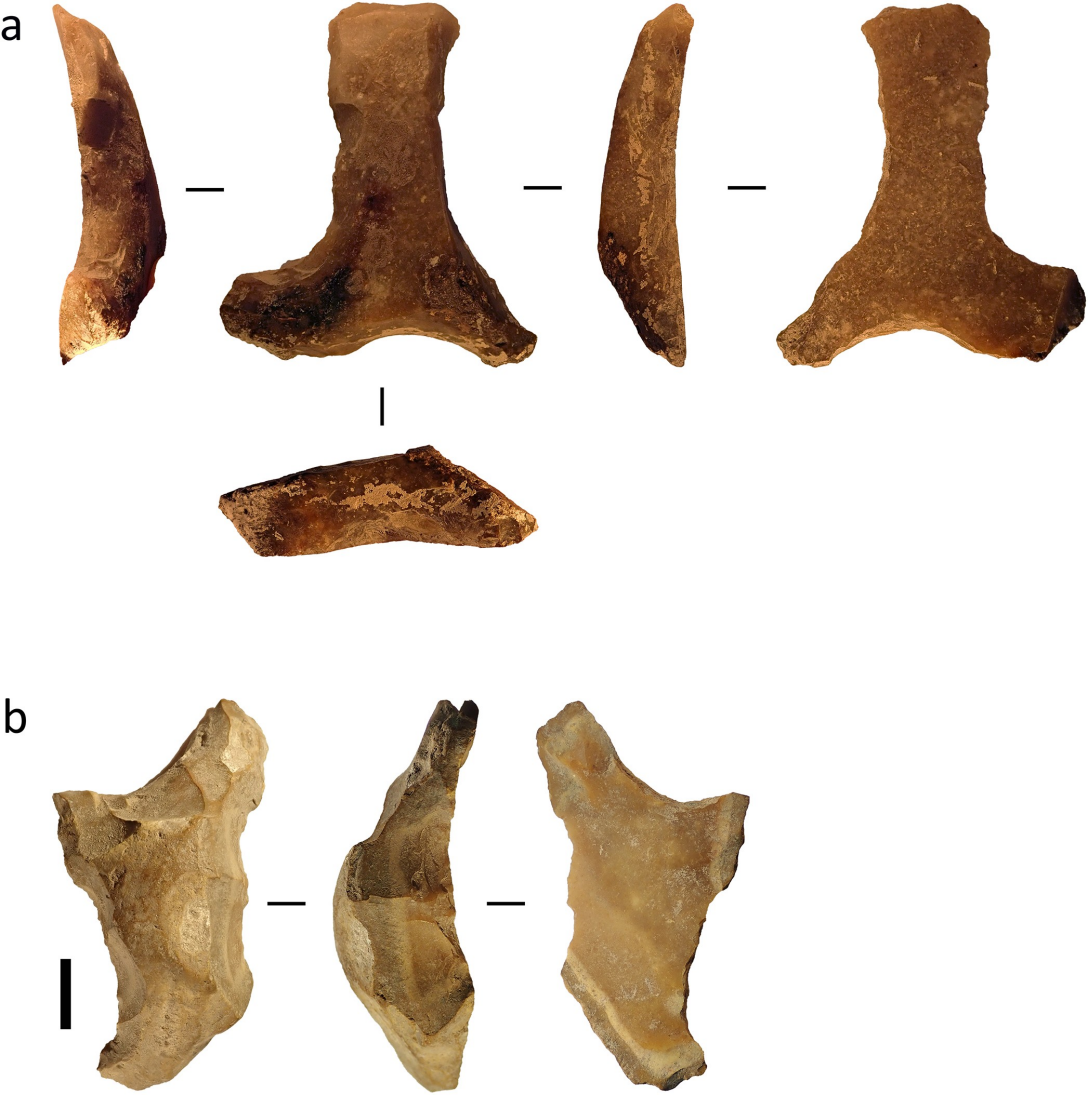
Heavily retouched flakes or ‘scrapers’ from Leang Panninge. (a) Artefact recovered from square S16T6, 182–202 cm below the surface. (b) Artefact recovered from square T17S6, 162–1720 cm below the surface. Scale bar = 1 cm.

Given this, it seems more practical for analysts to classify these particular objects as retouched artefacts unless future work can identify patterns in function or morphology. Among the assemblages in our study two heavily retouched flakes recovered from Toalean deposits at Leang Panninge show multiple deeply concave edges (Fig 2). These artefact resemble retouched stone artefacts recovered from the eastern Sulawesi cave site of Goa Topogaro, which usewear analysis has associated with bone point manufacture [89, 90], so it may be that the Panninge examples served similar function. Usewear analysis could test this.

Shell scrapers are also frequently reported among Toalean assemblages [e.g. 28, 69, 91]; however, it is unclear how these were distinguished from trampled bivalves or those that have been processed for food extraction. Various studies have shown that cultural modification of shell scrapers is often difficult to distinguish from taphonomic damage based on macroscopic features alone [e.g. 92, 93]. Again, it is suggested that dedicated analysis is required before these can be reliably recognised as a Toalean artefact class.

#### Bipolar artefacts [*artefak bipolar*]

**Fig 3:**
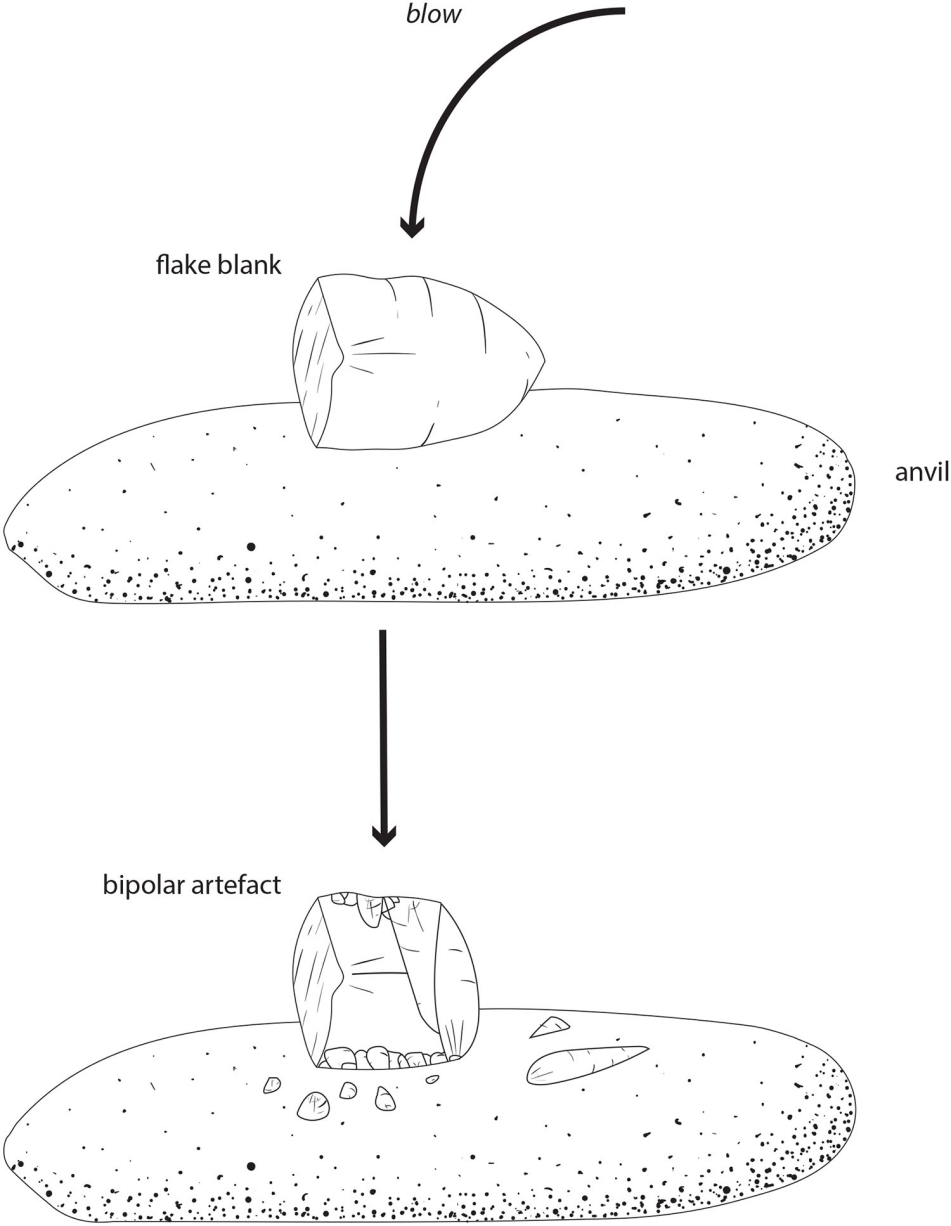
Bipolar reduction. Bipolar reduction involves bracing a core on an anvil and striking it from above, a process which can initiate fracturing at both points of contact.

Toalean assemblages sometimes include bipolar artefacts; however, these are not common nor specialised enough to yet suggest a specialised industry such as we see in the Pleistocene assemblages of the neighbouring island of Alor [94]. Bipolar reduction occurs when a stone is braced on an anvil and struck from above with a hard percussor, splintering it into sharp pieces [95]. Bipolar artefacts are also called scalar cores, fabricators, or *outils écaillés* [96]. While bipolar pieces may not always be recognised and may therefore be underreported [97], the process typically creates pieces with diagnostic features including crushing at both points of impact (i.e. the anvil and the percussor), stacked compression rings, and wedging initiations [98]. Bipolar reduction employs a different set of technical gestures to the freehand percussion process, and the kinaesthetics are more similar to that of backing and truncating [83] (Fig 3).

Bipolar reduction was a common technique used throughout the world, and may be linked to the earliest development of stone tools [99, 100]. In ISEA, bipolar reduction has been reported at a number of Pleistocene and Holocene sites [e.g. 83, 94, 101], including the Pleistocene assemblages in South Sulawesi of Leang Bulu Bettue [34], Leang Sakapao 1 [33, 102], and Leang Burung 2 [35, 103]. The technique was also practiced in the historic period in Papua New Guinea [96].

**Fig 4:**
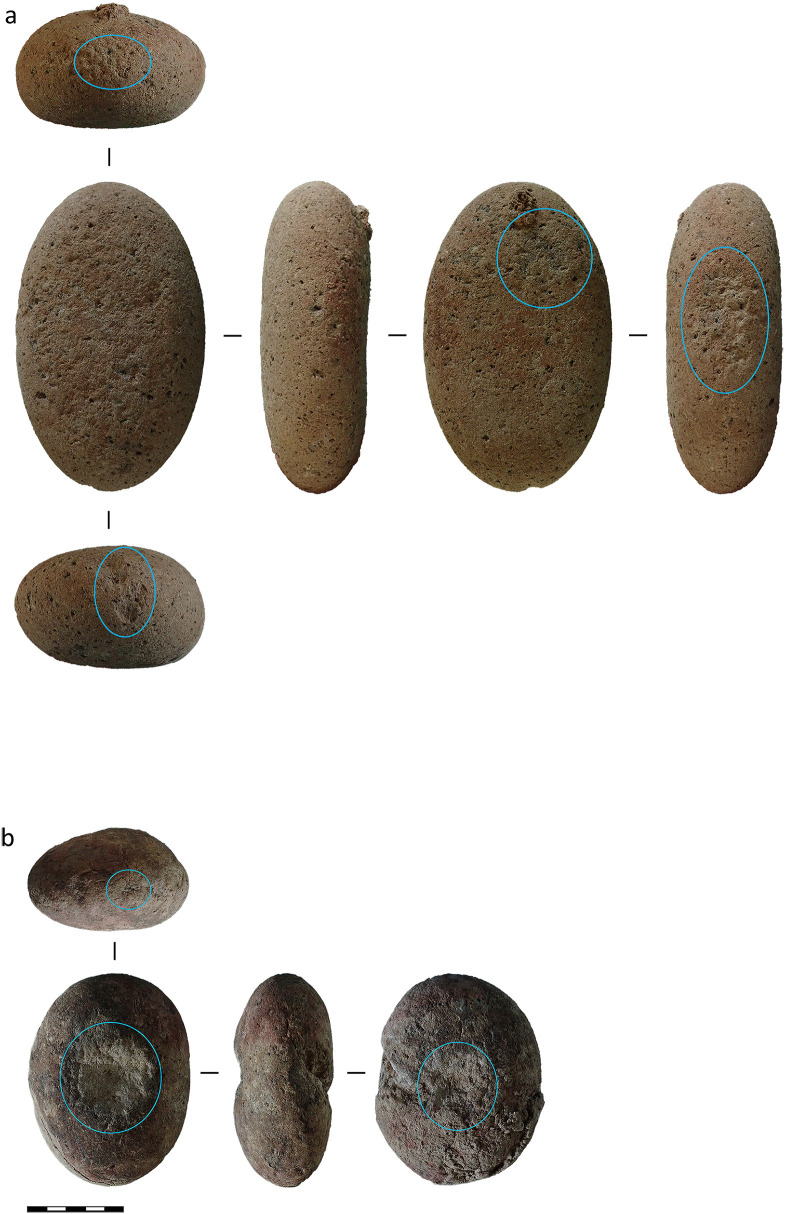
Anvils and hammerstones from Leang Pajae, Test Pit 1. The battered areas on these river cobbles are circled in blue. (a) Cobble recovered 0–10 cm below the surface, within Layer 1. (b) Cobble recovered 50–60 cm below the surface, Layer 3. Associated finds suggest these are Toalean deposits [unpublished], though (a) originates from a layer which includes mixed Neolithic finds. These two artefacts are more likely to be associated with bipolar reduction than nut or seed processing [105]. Scale bar = 5 cm.

Bipolar reduction was also employed during the Toalean period. For example, bipolar pieces were recovered from excavations at Ulu Leang 1 (*n* = 70, 1.3% [28]), Leang Burung 1 (*n* = 15, 0.1% [8]), Batu Ejayya 1 (*n* = 2, 0.1% [8]), Balang Metti (*n* = 3, < 0.1% [53]), and from a recent survey of the Batu Ejayya complex including excavations of Pangnganikang (previously Batu Tuda, *n* = 5, 2.2% [69, 104]). We recoded 27 bipolar artefacts at Leang Pajae (2.0%, unpublished). Occasionally bipolar artefacts were used as blanks for Maros point production, with one example recovered from Leang Rakkoe [32], in the Bomborro valley, and a second from Leang Pajae (Fig 15). Bipolar pieces in Toalean assemblages were often made on freehand-struck flake blanks [69], perhaps using the side and centre of large, rounded river-stones as a hammerstone or anvil (Fig 4) [105]. Bipolar artefacts therefore make up a small portion of Toalean assemblages, and while persistently present across many sites, bipolar reduction does not appear to have been a dominant or specialised industry during the Toalean.

### Diagnostic Toalean artefacts

#### Backed microliths [*mikrolit berpunggung*]

Backed microliths are small flakes with abrupt retouch along one or more margins. While the exact definition of a ‘microlith’ can vary widely [see 106], backing is a form of retouch usually achieved by placing a flake on an anvil and striking down onto one margin of the flake with a hard-hammer, similar to bipolar flaking, to remove a series of tiny flakes along one or more margins. This produces a linear series of scars at almost right angles to the flake face. In some instances backing was done with a pressure technique [107]. It is often assumed that backed artefacts were produced for hafting singularly or as composite tools [e.g. 108–110], similar to preserved examples including the hafted arrows of Lushult and Rönneholm, Southern Sweden [111], the VI-XII Dynasty arrows of Naga-ed-Der, Egypt [112], or the historic use of backed stone and glass microliths as arrowheads by San groups in South Africa and the Kalahari [113]. However, as yet there is no direct evidence for hafting technology during the Toalean period of South Sulawesi.

**Fig 5:**
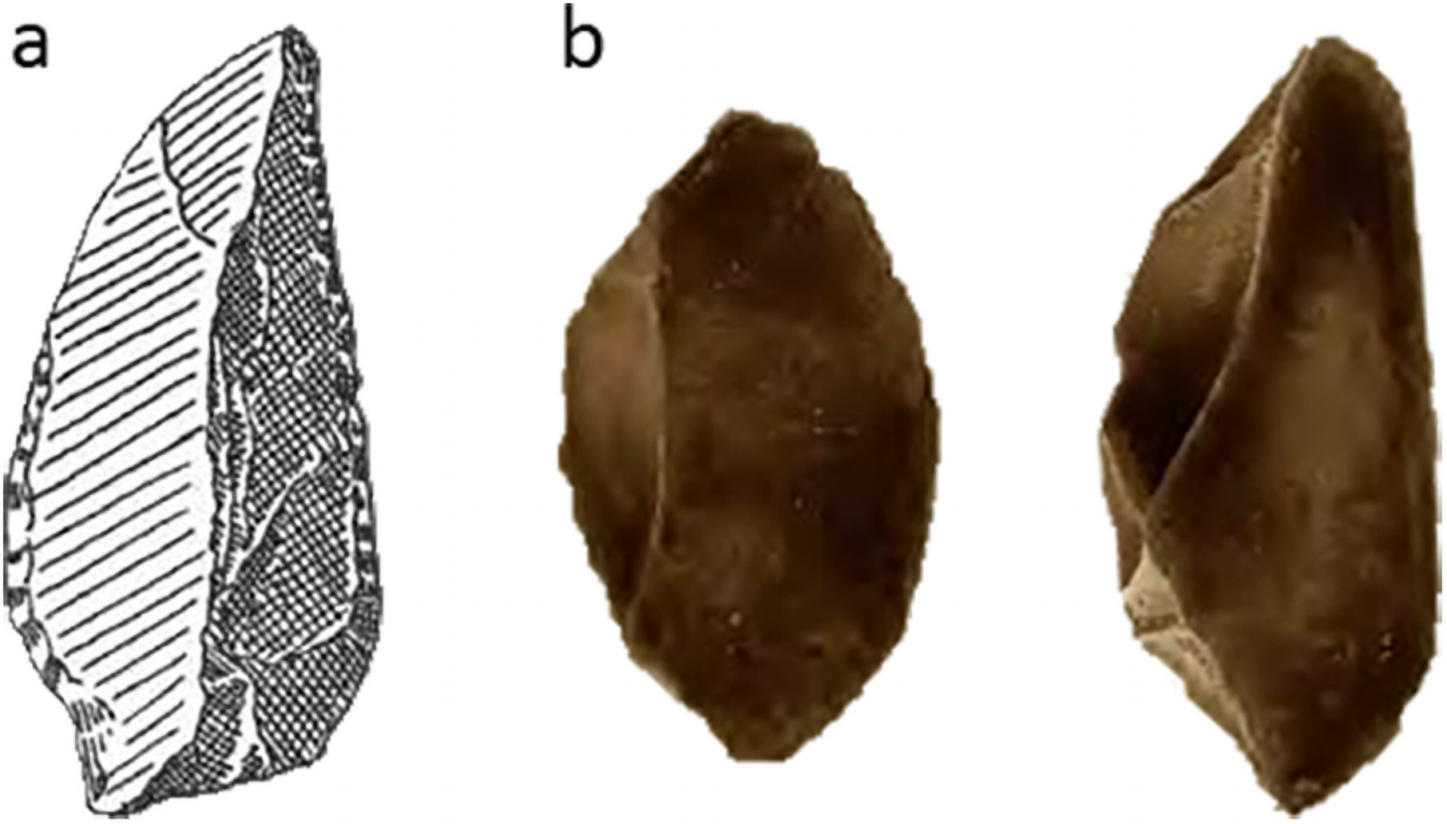
Early reports of backed microliths in Indonesia. (a) Backed microlith reported by Bandi in 1951 from Ulu Tjanko, Java [adapted from 118 Fig 8.1]. (b) Toalean backed microliths from Lamoncong, published by Sarasin and Sarasin in 1905 [adapted from 23 pl. 1.6 & 1.8].

Occurrences of backed artefacts are widespread through Asia, Australia, Africa, and Europe. The oldest known backed microliths come from the Twin Rivers area of Central Africa, where associated U-series dates suggest an antiquity of 200–300 ka [e.g. 114]. In some areas of Australia, backed artefacts may be Late Pleistocene in age [115, but see 116, 117], although most examples have been dated to the Holocene. In Indonesia, undated backed microliths have been reported from the Bandung Plateau of Java, as well as Danau Kerinci and Ulu Tjanko in the Jambi Provence of Sumatra [17, 85, 118], although the sketches provided in the original document [118] are unconvincing (Fig 5a). Backed microliths are prolific throughout Toalean assemblages [51, 85], contrary to a recent claim that backed microliths do not occur in the Island Southeast Asia (ISEA) region [108].

The earliest known reference to backed microliths in South Sulawesi, and indeed Southeast Asia, comes from an image published in Sarasin and Sarasin’s 1905 report on the natural history of Sulawesi [23] (Fig 5b), although the authors did not appear to recognise them as such [24 p. 27]. Backed microliths play an important role in attempts to define the Toalean, in that they have only been reported from Toalean special range and temporal period, and, further, form part of the basis for Bulbeck’s model of two Toalean entities [e.g. 28, 48]. Recently, backed microliths have been reported from Balang Metti, in the Bone regency, the first reported instance of this technology from a highland site [53, 119] (however see below for a second instance, at Leang Panninge).

**Fig 6:**
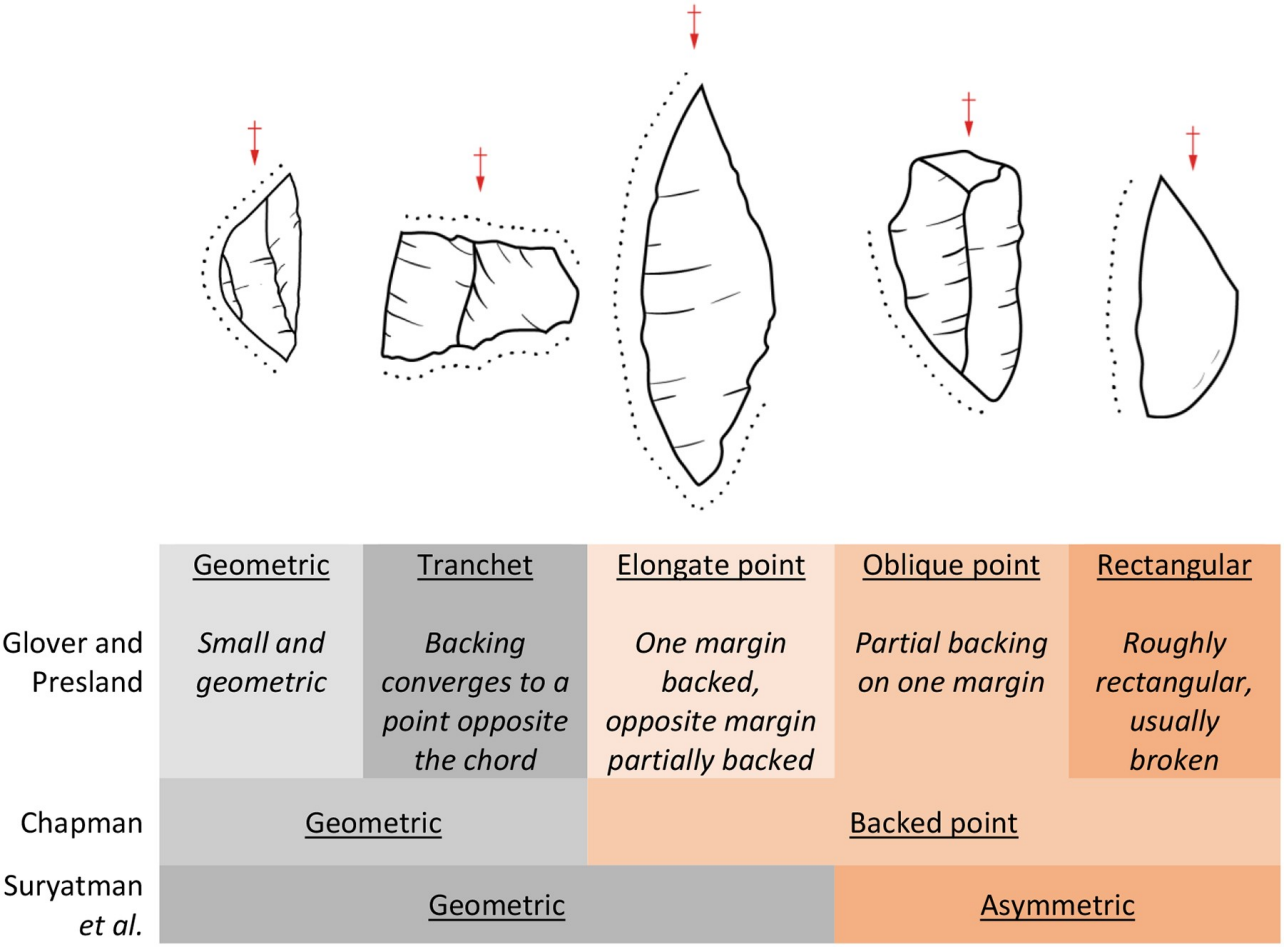
Different classification systems used on the backed microliths recovered from Toalean assemblages [8, 53, 85]. Red arrows indicate percussion axis. Artefact variation adopted from Glover & Presland [85 p. 19].

Several models have been used to subdivide the Toalean backed microliths; however, no one model has become unanimous (Fig 6). Based on an analysis of 262 backed flakes from an excavation of the Toalean deposits at Ulu Leang 1, in the Maros regency, Glover and Presland [85] subdivided the backed microliths into five types based on the distribution retouch and the authors’ intuitive observations of morphology [9, 28, 85]. In contrast, Chapman [3, 8] followed an approach popularised by McCarthy [120] and recognised only two forms of backed microlith among the assemblage she examined from Leang Burung 1: backed points and geometric microliths (or sometimes ‘bipoints’, see p. 46 of [3]). Some recent works by Suryatman [e.g. 53, 57] follow a similar system to Chapman’s, but her justifications for these primary divisions remain untested. We suggest that a more quantitative approach should first be adopted to characterise the morphological continuum in backed microlith shape, such as the Backed Artefact Symmetry Index (BASI) [121], a modified from the Maximum Width Position metric [122].

The reduction process employed on the backed microliths of Leang Panninge, in the highlands of the Maros regency, is given special attention here as these artefacts display a combination of features including ventral retouch not reported on other Toalean microliths. While a thorough analysis of the lithic assemblage at this cave site is ongoing, initial reports have identified a rich Toalean assemblage including Maros points, backed microliths, and osseous points [123, 124], and possible evidence for early management of Suidae at the site [29, 125].

**Fig 7:**
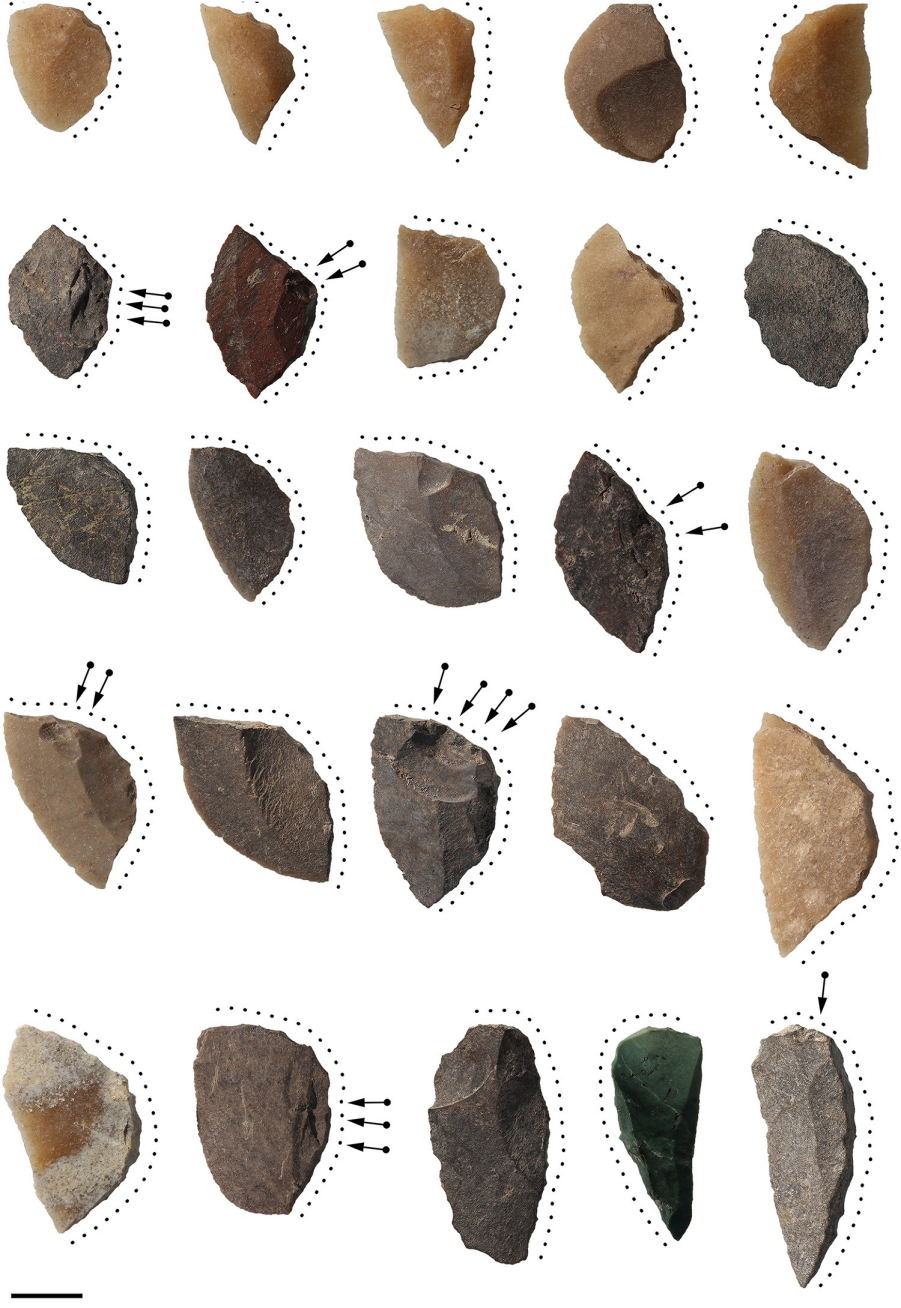
Leang Panninge backed microliths. The microliths are oriented with the percussion axis vertical, the dorsal surface facing up, and the flake blank’s proximal end towards the top of the page. The extent of backing is indicated by dotted lines, and diacritical marks denote the retouching across the visible face of the microliths. Scale bar = 1 cm. Image credit: Nur Ihsan.

Leang Panninge contains dense Toalean assemblages, and 138 unbroken backed microliths were recovered from the upper 150 cm of two 1m x 1m squares excavated in 2015 (squares S17T6 and S16T6) [124, 126]. These microliths were backed along one lateral margin, and backing extended across the platform in 118 instances (86%). In 121 cases (88%) the backing occurs on the right lateral margin, when orientated with the proximal end to the top and the dorsal face up (Fig 7). Furthermore, 77 (58%) of the backed microliths are ‘lozenge’ shaped in plan-view [after 127], with the backing forming a continuous curved edge opposite an unmodified curved lateral margin or ‘chord’. This lozenge shape is less pronounced in more elongated and asymmetric backed microliths (Fig 7). In many cases these microliths have no dorsal scars, and appear to have been struck from the ventral face of a larger flake blank, occasionally even removing the flake blank’s bulb of percussion. Striking microliths from the ventral face of a larger flake may have enhanced the curvature of the chord observed on many of the backed microliths. The regularity of lozenge morphology is presently only known from Leang Panninge, although it does resemble one of the *einchneidige messer* (single-edge knives) the Sarasins illustrated from Lamoncong (previously Lamontjong) [23] (Fig 5).

**Fig 8:**
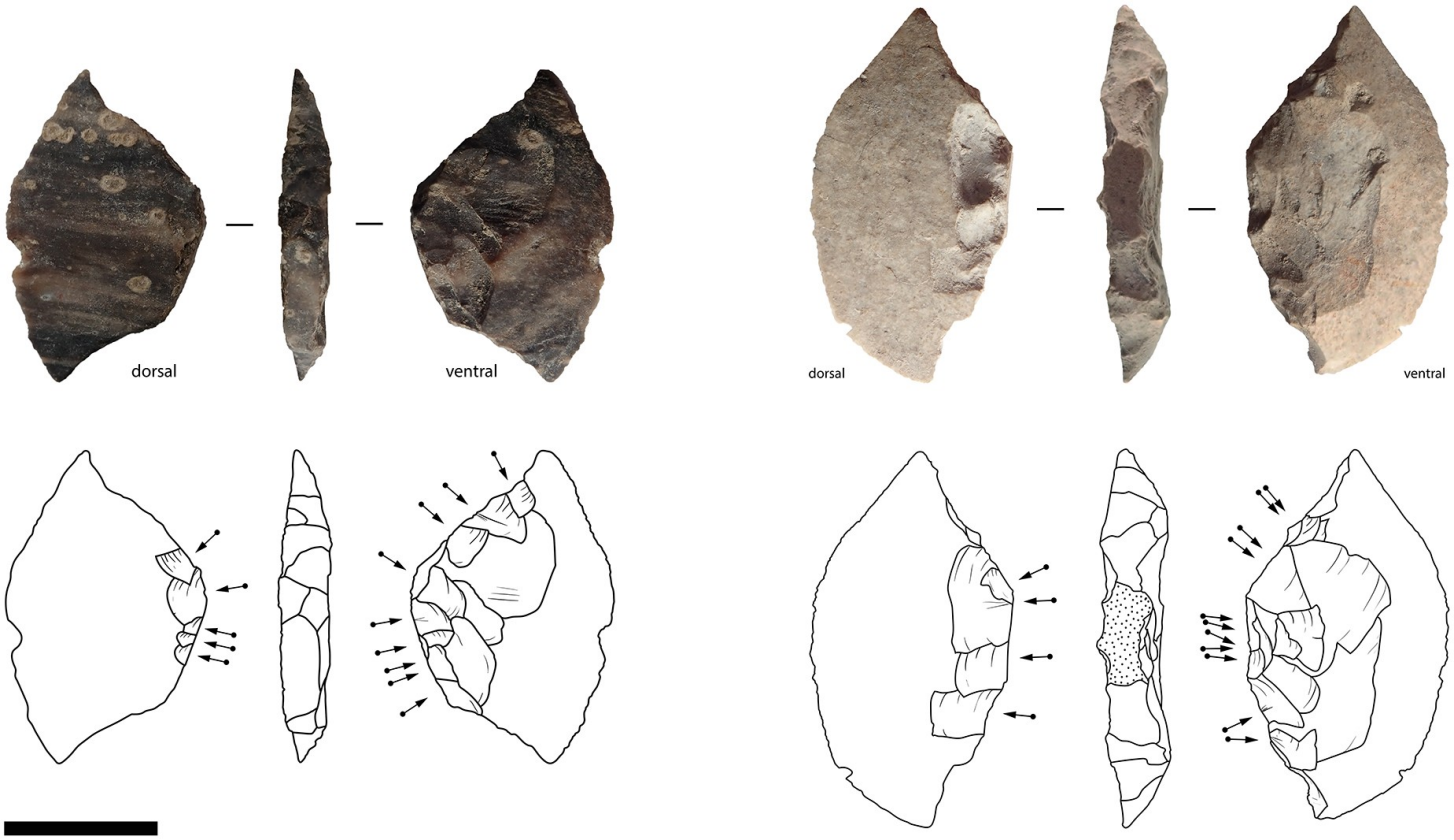
Retouched backed microliths. Backed microliths from Leang Panninge with extensive retouch across the dorsal and ventral faces. Retouching was done after the artefacts were backed, using the backed edge as the striking platform. Scale bar = 1 cm.

On 40 (29%) of the backed microliths from Panninge, small retouching flakes were also struck across the dorsal and/or ventral face of the microlith using the backed edge as the platform (Fig 8). Some of these non-invasive retouch scars terminate in a hinge or step, as they were struck into an area of low mass. Impact fracture or hafting damage can be ruled out as the cause, as the scars are well-spaced and the opposite chords of these microliths show no macroscopic damage or crushing from usewear. It is unclear why this additional retouch was done, but the process thinned the backed side of the microlith, perhaps assisting hafting. Such technological observations may be of use in future investigations into intersite and regional variations in production approaches.

#### Sawlettes [*gergaji kecil*]

**Fig 9:**
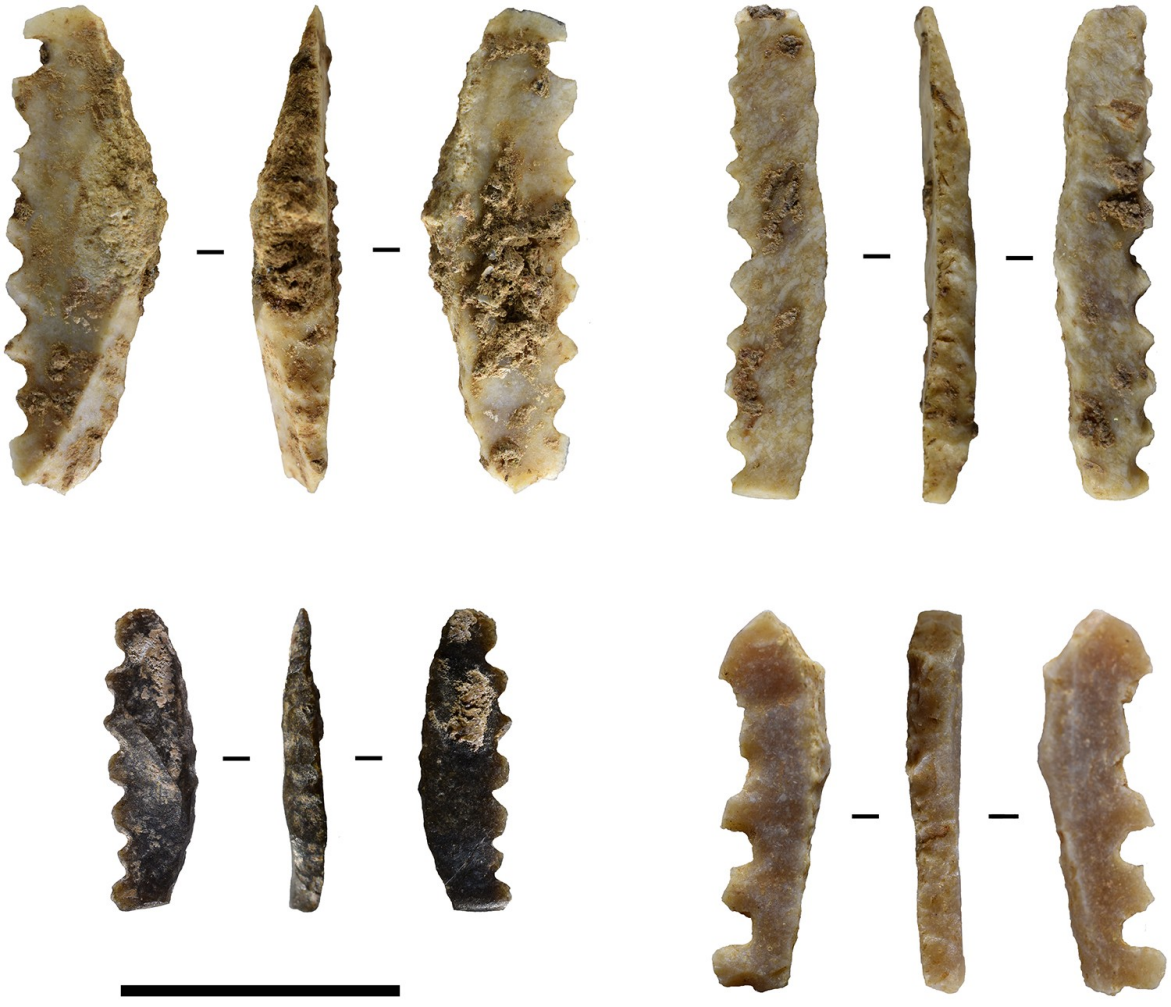
Leang Bulu’ Sipong sawlettes. Toalean ‘sawlettes’ recovered from the upper 40 cm of Leang Bulu’ Sipong 1, trench T9S1. Scale bar = 1 cm.

A growing collection of tiny, slender, backed and denticulated microliths suggests that it may be practical to recognise these artefacts separately. Twelve narrow, denticulated and backed microliths were recovered from the Toalean site of Leang Bulu’ Sipong 1 (unpublished excavation in 2018, extending on the 2017 excavations of [128] where the site is referred to as Bulu Sippong 4), that are morphologically and technologically distinct from the backed microliths described above. Leang Bulu’ Sipong 1 is a low-lying limestone cave situated at the foot of a limestone karst inselberg on the coastal plains of the Pangkep regency, less than 100 m from the Late Pleistocene rock art site Leang Bulu’ Sipong 4 [38]. The following typological description was compiled from 12 examples of this lithic artefact recovered from Leang Bulu’ Sipong 1 as well as two from Leang Jarie [57]. Ten of these are broken transversely, and measurements are taken from complete artefacts. Dubbed here ‘sawlettes’, these artefacts were made on tiny blade-like flakes, often with one or two parallel dorsal arises (Fig 9, Table 1). The blanks are backed along one margin, while the opposite margin is finely denticulated or serrated. These narrow denticulations were carefully formed using a thin pressure flaker of unknown material–possibly the edge of another flake. Apart from the transverse breaks and some broken teeth, which may have occurred during manufacture or post-deposition, no signs of usewear or gloss were observed under 15 x magnification. The Leang Bulu’ Sipong 1 finds are currently undated, but were clearly associated with Toalean artefacts including Maros points and osseous points, while the sawlettes at Leang Jarie were recovered from layers that included charcoal samples dated to 2850–2750 and 7870–7750 calBP [57]. The function of these sawlettes remains unclear.

**Table 1:** Dimensions of the 14 sawlettes recovered from Leang Bulu’ Sipong 1 and Leang Jarie.

Attribute	*n*	Range	Mean	Standard deviation
Length (mm)	4	10.86–17.53	15.07	0.63
Width (mm)	14	3.24–5.67	4.06	0.69
Thickness (mm)	14	1.20–2.83	1.77	0.41
Weight (grams)	14	0.07–0.20	0.13	0.04
Sample denticulation depth (mm)	12	0.53–1.59	0.81	0.29
Sample denticulation spacing (mm)	12	1.28–2.83	1.99	0.39

Such artefacts have rarely been reported in earlier studies, and it is likely that they were overlooked in previous excavations as they are quite small and relatively rare. Of the 10,554 artefacts Chapman analysed from Leang Burung 1 [3], she describes three “tiny, slender blades with one margin denticulated unifacially to form a point with the other margin, which has very small abrupt unifacial retouch along 50% or less of its length from the proximal end” [3 p. 82] in the upper levels of Trench B. These artefacts measured 13–17 mm long by 3-5mm wide and 1–2 mm thick. Chapman suggests they may have been dart points and classifies them under ‘miscellaneous tools’ [3 pl. 4.7.4, p. 82B, 8]. Bulbeck *et al*. [20 p. 95–96] suggest that ’backed and denticulated microliths’ were likely spear barbs, and it is possible this refers to the Bulu’ Sipong sawlettes. It is unclear at which sites these artefacts were observed. Similarly, Glover [28] lists ‘denticulated bladelets’ among the Toalean tool types, but without any further information or illustrations. It is hoped that by describing this variety of backed microlith here these artefacts will become better recognised and studied.

**Fig 10:**
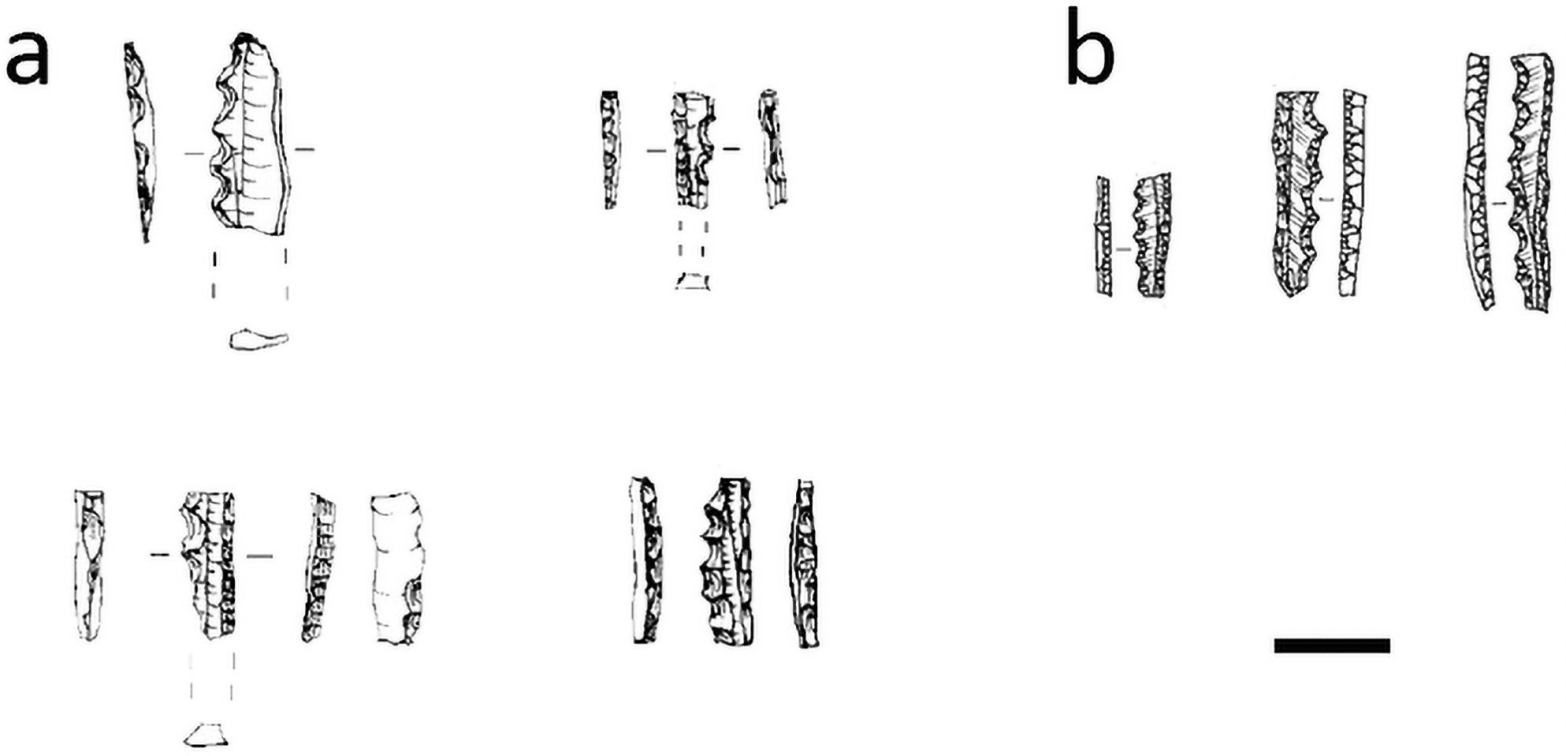
Microdenticulates from Upper Paleolithic Europe. These small Gravettian artefacts closely resemble the sawlettes of Toalean Sulawesi. (a) Adapted from [129 p.3 Fig 2]; (b) adapted from [130: p. 161, Fig 6.8–6.10]. Scale bar = 1 cm.

The Bulu’ Sipong sawlette resembles an Upper Palaeolithic tool type of Central Europe often called a microdenticulate [e.g. 129 Fig 2.1–2.4, p.3, 130 Fig 6.8–6.10, p. 161]. Like the South Sulawesi artefacts, these artefacts are tiny backed blades with fine denticulations down the chord opposite the backed margin (Fig 10). The European tools were produced in the Austro-Moravian-south Polish corridor during the Pavlovian stage of the early Gravettian period, around 30–25 ka ago [131]. Given this temporal and special separation, the similarity between these Toalean and European serrated microliths is a clear case of technological convergence in the archaeological record [132], potentially functionally driven. We propose the name ‘sawlette’ is more appropriate for the Toalean artefact type than microdenticulate, to avoid implying a cultural connection. Furthermore, ‘microdenticulate’ is a broad term that can also be applied to macroblades and flakes with small denticulations [e.g. 133–135]. Other terms for these Gravettian tool include ‘denticulated backed microblade’, ‘backed microsaw’, ‘*mikropilka*/*mikropilka na čepel s otupeným bokem’* (from Czech: ‘microsaw/backed blade microsaw’), and ‘microdenticulated backed microblade’ [136, 137].

#### Maros points [*lancipan Maros*]

**Fig 11:**
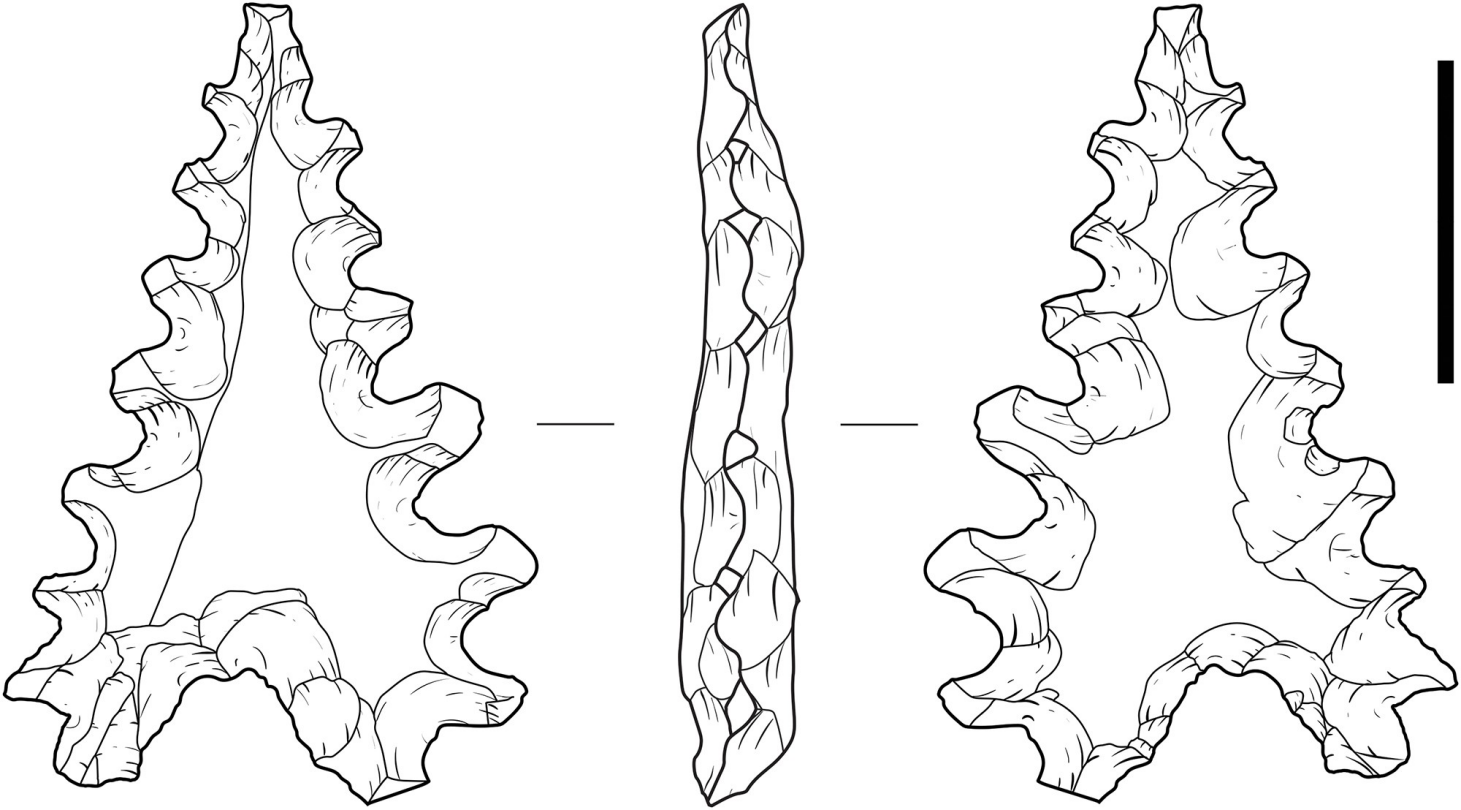
A classic Maros point. Point recovered from Leang Pajae, Test Pit 1, 20–30 cm below the surface. Scale bar = 1 cm.

Maros points are small stone points unique to the Toalean period and to the South Sulawesi region. They are roughly triangular in plan form, and have two defining features: an indented base and/or shallow to deep denticulations on two margins which converge at a tip (Fig 11). However, researchers disagree over which features are integral in defining the type. The Toalean points were first reported in the early 1900s at excavations in Lamoncong, where the Sarasins described them as ‘arrowheads’ with saw teeth [23 p. 14]. Van Heekeren likewise called them *gevleugeld* or *getande pijlpunten*–winged or toothed arrowheads [28, 138 p. 92]. The name ‘Maros point’ was proposed in 1970 by Mulvaney and Soejono [55], after the Maros regency of South Sulawesi in which the most Toalean research had been conducted at that time. They described the artefacts as hollow-based points to avoid implying function, pointing out that “there is no basis in fact for calling it an arrowhead” [55 p. 171]. However, they make no mention of serrations or denticulation being essential to the definition.

Chapman is more specific, defining Maros points as those with a deep basal indentation (>2 mm deep) with either denticulation and/or linear retouch along the margins, while all other retouched points are classified as ‘miscellaneous points’ [3, 8]. In contrast, Glover and Presland [9, 85] split the Maros point type into four sub-types based on the method of margin retouch (denticulate, Helwan retouch/oblique bifacial, oblique unifacial, and backed–though note that the illustration provided represents steep retouch rather than backing [85 Fig 3, p. 192] suggesting that the technique may have been misidentified), speculating that these variations may relate to the type of game targeted and that poison was applied to certain edge types [85], although there is no residue analysis to support this. Under this system, Maros points may or may not have a hollow base and are seen to grade into the tranchet forms of backed microliths. This differs again from Bulbeck [50], who describes Maros points as only those with both denticulated margins and a hollow base, a distinction also followed by Suryatman [e.g. 57, 104]. Bulbeck adopts the term ‘Malindrung point’, after Hakim [139], for denticulated points without a basal indent, based on samples collected near the village of Mallinrung on the Upper Walanae River of the Bone regency [20, 50]. Bulbeck also labels retouched points with neither denticulations nor a hollow base as ‘pirri points’, after an Australian artefact type of that name [7, 50].

**Fig 12:**
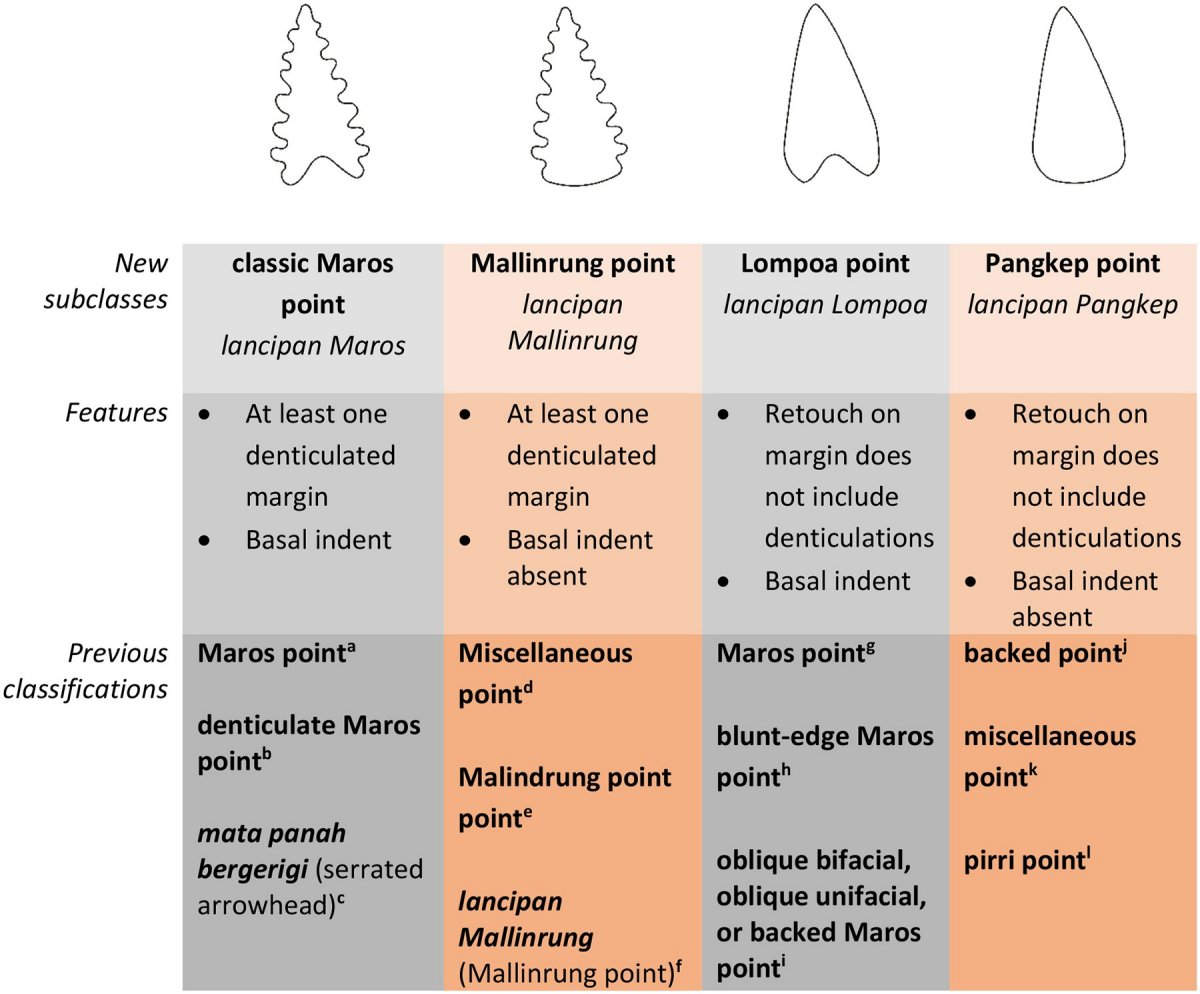
Subclasses of Toalean retouched points. ^**a**^ [e.g. 8, 50, 55, 57, 123, 141], ^**b**^ [28, 85], ^**c**^ [25], ^**d**^ [8], ^**e**^ [20, 50], ^**f**^ [139], ^**g**^ [8, 55], ^**h**^ [28], ^**i**^ [9, 85], ^**j**^ [3], ^**k**^ [8], ^**l**^ [50].

**Fig 13:**
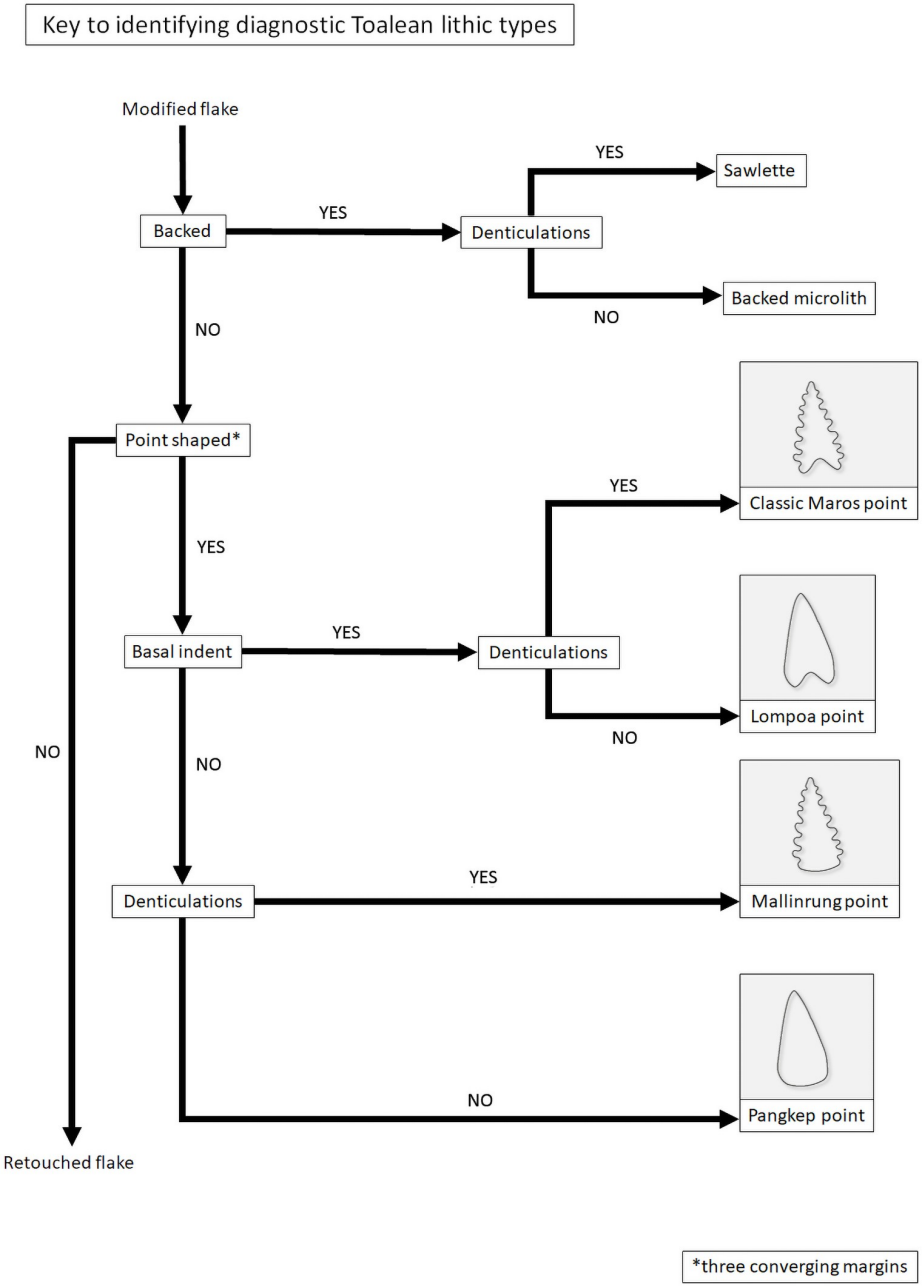
Key for identifying the defining Toalean lithic classes.

It is clear then that the current system for classifying Maros points is inconsistent. For clarity and consistency we suggest a system that recognises all four possible combinations of the two key variations, that is an indented base and denticulated margins, regardless of retouch type or direction (Fig 12). These four variations we propose calling: the classic Maros point which has both defining features; Mallinrung points with denticulated margins and no basal notch [after 20, 139]; Lompoa point with a basal notch and non-denticulate linear retouch to form a point; and Pangkep point [previously ’pirri points’ in 50]. These can be identified by following the flowchart provided in Fig 13. The term ‘pirri point’ is not adopted here as pirri points are specifically an Australian tool type and are, for the most part, exclusively unifacially flaked–although some have been bifacially flaked across the bulb of percussion–and the scars are typically invasive and remove much or all of the original dorsal face [e.g. 140], which as the following section will demonstrate is not seen on any of the Maros point subclasses. All four variations of Maros point can be distinguished from backed microliths in their general point-like morphology, unifacial or bifacial retouch which occurs on more than one margin, and while the retouch on the points may be steep it is not abrupt enough to classify as backing.

#### Maros point technology

**Fig 14:**
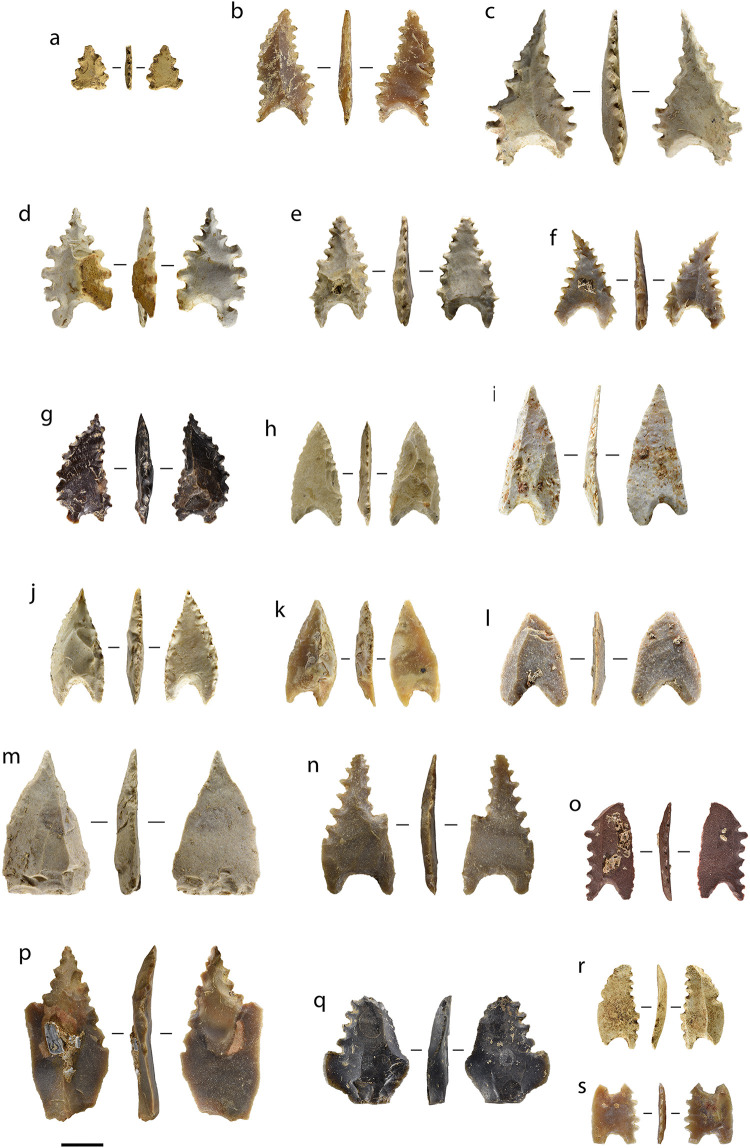
Toalean points. (a)–(g) Classic Maros points; (h)–(i) Lompoa points; (m) Pangkep point. (n), (o), (r) Appear to be unfinished classic Maros points; (p)–(q) Mallinrung points. Some artefacts may not be clearly classifiable, such as the double-based example (s). Scale bar = 1 cm.

This model was developed and then tested by applying it to the 212 retouched points recovered from excavations at Leang Bulu’ Sipong 1 (*n* = 146), Leang Pajae (*n* = 47), and Leang Rakkoe (*n* = 9), and surface collections from Leang Lambatorang (*n* = 7), Leang Lampoa (*n* = 1), and Tallasa (*n* = 1) (Fig 14). Of these, around half were broken (*n* = 108, 51%), often during production, from heat damage or from trampling. Notable pieces among these include one classic Maros point with what appears to be impact damage at the tip, and another exhibits shallow denticulations across the basal notch.

Some 72% (*n* = 152) of the point-like artefacts can be classified under the system proposed above. Seven of the analysed points are classified as Mallinrung points, 102 are classic Maros points, 32 are Lompoa points, and 11 are Pangkep points. Of the remaining 60 unclassified pieces, 46 are too damaged to show all of the diagnostic features, one artefact has a basal hollow at each end (Fig 14s), and another is a flake with four denticulated margins and no base; these could be classed as ‘unidentified points’ and ‘miscellaneous’ respectively. Finally, the remaining 12 objects in our study were very minimally modified, and may be manufacturing rejects or ‘incomplete points’ [following 142].

Based on the 152 classified Toalean stone points from the Maros and Pangkep regencies, the following model for Toalean point production was developed. The first stage of production involved selecting a small, fairly flat flake blank for reduction. These may have been deliberately produced by setting up platforms to target flat areas of the core face, but to date analysis of cores has not documented this process. At this stage it seems equally likely, and more parsimonious, to assume that flakes were selected by searching through the debris from core reduction. This is supported by the identification of three cases at Leang Bulu’ Sipong 1 where flakes with usewear gloss were recycled as blanks for Maros point production. Flake blanks were produced by direct percussion, occasionally with prior overhang removal (*n* = 2, 1%), and often struck down an unpronounced dorsal ridge (*n* = 98, 64%).

**Fig 15:**
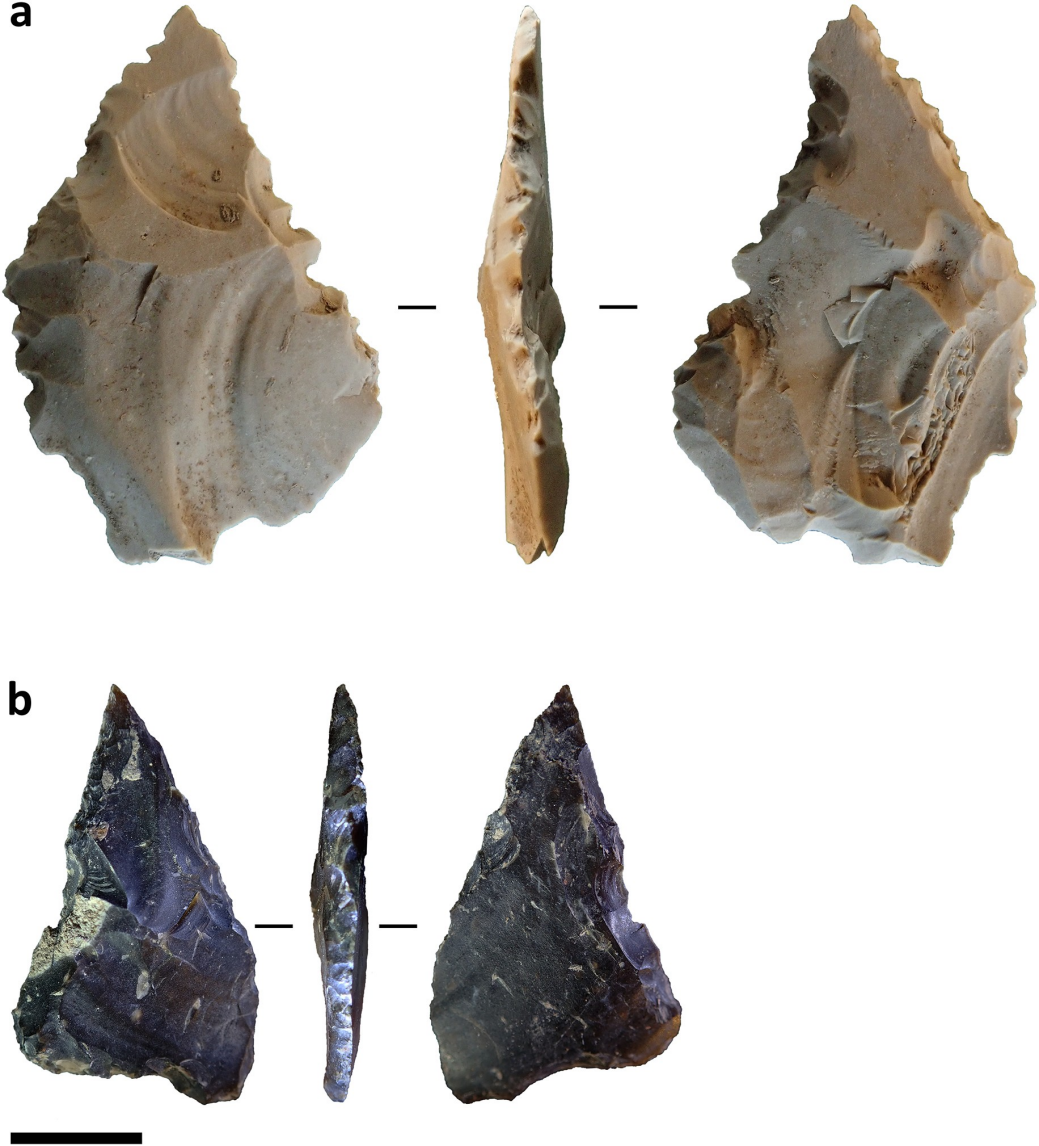
Lompoa points made on a bipolar flakes. (a) Artefact recovered from Leang Pajae, Test Pit 2, 0–10 cm below the surface. (b) Artefact from Leang Rakkoe, Test Pit 1, 1–10 cm below the surface [32]. The pressure retouching scars intrude into the scars created in bipolar reduction. Scale bar = 1 cm.

Where a basal hollow was added (i.e., the classic Maros points and Lompoa points), the second stage often involved forming the base before the margins were modified. This is evident by the occurrence of pieces with well-formed hollows and unfinished denticulations, and through the diacritical analyses of the order of flake scar overlap which identified denticulation scars that intrude into basal flaking scars. The reduction process was highly variable, however, and in some cases the basal notch was formed after the edges were flaked [57]. The base was hollowed out with small, neat, non-invasive pressure retouching, usually at the thicker, proximal end of the flake blank (*n* = 101, 66%), perhaps with the aim of removing the bulb of percussion [143]. In 8% (*n* = 12) of analysed cases the base was located at the distal end. The propagation axis could not be determined on 40 of the analysed sample however, most of which were broken. In one case the base was made on the lateral margin of the flake–this is a point made on a bipolar blank, from Leang Rakkoe (Fig 15).

**Fig 16:**
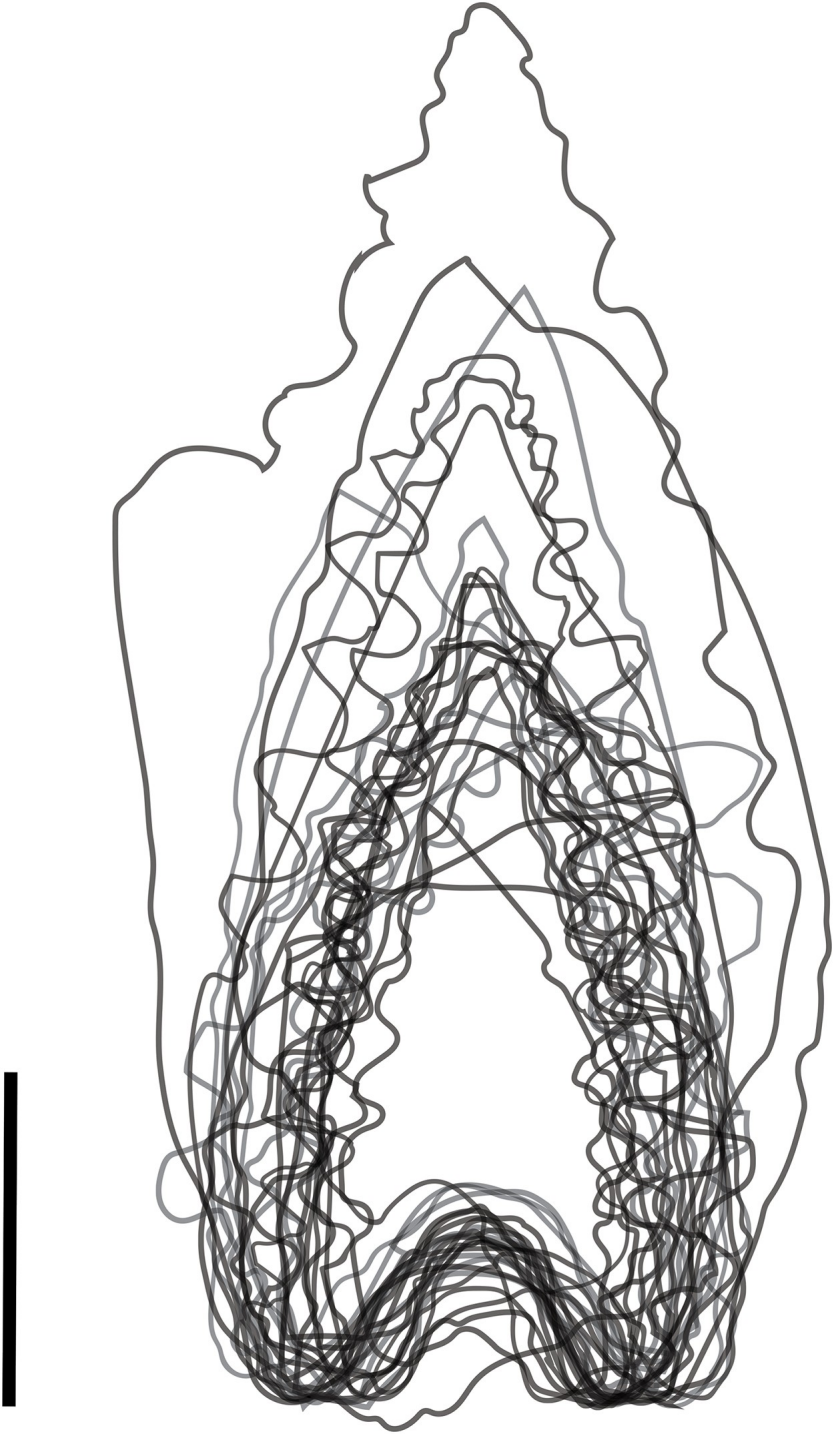
Diversity in Maros point shapes. The outlines of 26 Maros and Lompoa points demonstrate the high variation of morphology of the margins and relatively consistent basal shape. Scale bar = 1 cm.

The final stage involved modifying the edge, either with linear retouch (Lompoa points and Pangkep points) and/or pressure-flaked denticulations (classic Maros points and Mallinrung points). As noted by Forestier *et al*. [144], edge modification on Maros points was minimal. This is because thin blanks are necessary for producing fine edge denticulations in the absence of edge thinning through invasive flaking. This minimal modification leads to a wide variation in margin shape (Fig 16), although the morphology of the basal indent is somewhat consistent (Fig 16, Table 2), suggesting the base was formed to match a hafting system.

**Table 2:** Toalean points measured in this study^a^.

Attribute	*n*	Range	Mean	Standard deviation
Length (mm)	94	10.80–37.06	25.24	4.67
Width (mm)	141	8.04–20.73	13.81	2.49
Thickness (mm)	146	1.52–5.46	3.34	0.89
Weight of complete artefacts (grams)	89	0.12–3.09	1.06	0.57
Sample denticulation length/depth (mm)	99	0.40–3.15	1.66	0.61
Sample denticulation spacing (mm)	92	0.92–4.87	2.97	0.74
Flake thickness at base of denticulation (mm)	84	0.58–1.93	1.17	0.32
Sample width of denticulation-scar platform (mm)	114	0.31–1.53	0.74	0.21
Max. retouching scar length (mm)	148	0.66–10.23	2.64	1.27
Max. retouching scar width (mm)	148	0.86–14.14	3.38	1.54
Width of basal notch (mm)	95	4.72–16.15	8.29	1.98
Depth of basal notch (mm)	97	1.01–8.41	3.82	1.17

^a^ Measurements are only taken from unbroken features.

**Fig 17:**
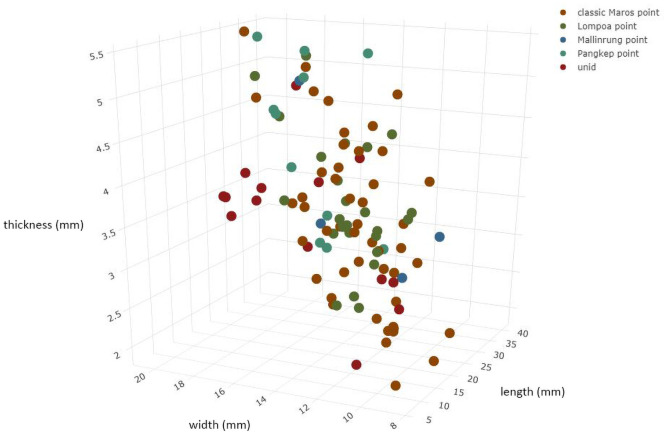
Maros point dimensions by type. 3D scatterplot of the dimensions of 102 unbroken point types, illustrating a similar range of sizes across all point types.

Edge work on both the base and margins was largely ‘non-invasive’, meaning that the flake scars do not extend to the centre of the face of the point [adapted from 12, see also 145], and as a result most of the dorsal and ventral features of the flake blank are still visible. Several points had linear retouched margins combined with denticulations running down only part of the margin, suggesting that when flake blanks were not sufficiently pointed they were retouched into shape before they were denticulated. This may suggest that the Lompoa point could be denticulated to produce a classic Maros point, and that the variety of forms may represent early stages of production–this is an avenue for future work as investigating intent and function fall beyond the scope of this study. The four variations of Maros points show the same range of dimensions, with no signs of grouping (Fig 17), and size and proportions cannot be used for classification. The range and variation of features in all Toalean points in our study are summarised in Table 2.

**Fig 18:**
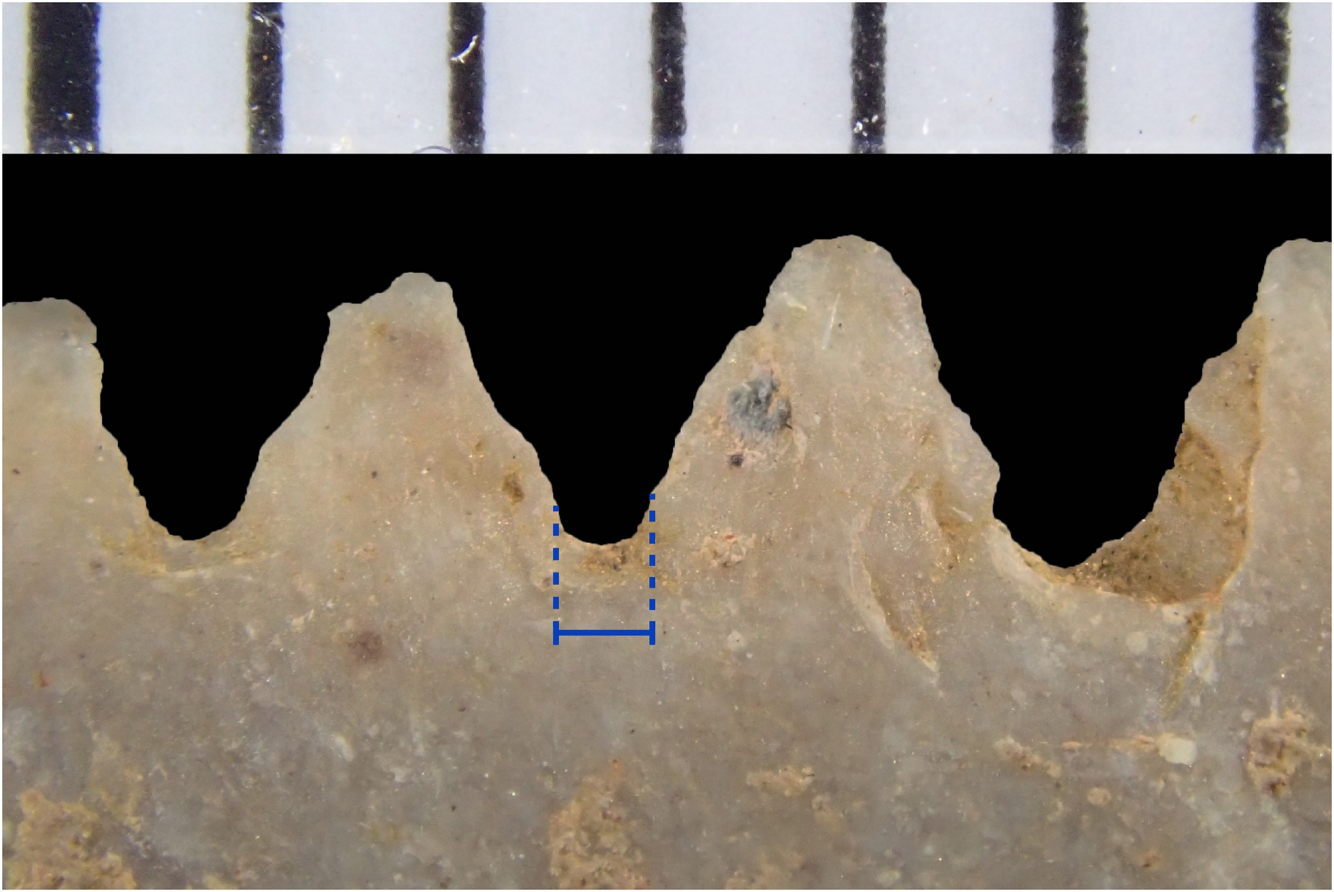
Denticulation scar platforms. The platform width of the denticulation scars (indicated by blue lines; see Table 2) was measured as a proxy for the maximum width of the tip of the notching tool. Scale bar is marked in 1 mm increments.

In order to study the possible tools used for pressure flaking the base and denticulations, the average width of the platforms of the denticulation or notching flakes [after 146] was measured from the scars they produced, as a proxy for the width of the tip of the tool used to form these denticulations (Table 2, Fig 18). Given that these are, on average, less than 1 mm wide (Table 2), it seems likely that the edge of another flake was used as an indentor to denticulate these artefacts. The Sarasins attempted to replicate Maros points by using the edge of another flake, which they claim was successful [23, also suggested by 147]. Ethnographic records show that stone has occasionally been used as a pressure flaker, as accounts from Point Barrow, Alaska, describe flint being used as the working end of hafted pressure flakers [148]. Titmus [146] successfully replicated serrations on North American-style points using the edge of another flake, although he found that this method was easier if the flake indentor was hafted into a handle. Titmus noted that the disadvantages of this method was that the flake serrator tool breaks easily, and can slide off the platform without initiating retouch [146]. The osseous points commonly found in Toalean sites would likewise be narrow enough at the tip to produce these denticulations, although this possibility is yet to be systematically tested (but see [149] for preliminary experiments with bone). Our own preliminary experiments suggest that other organics such as wood, shell, or bamboo are too weak to initiate fracture once whittled down to the requisite width.

#### Osseous points [*lancipan tulang*]

**Fig 19:**
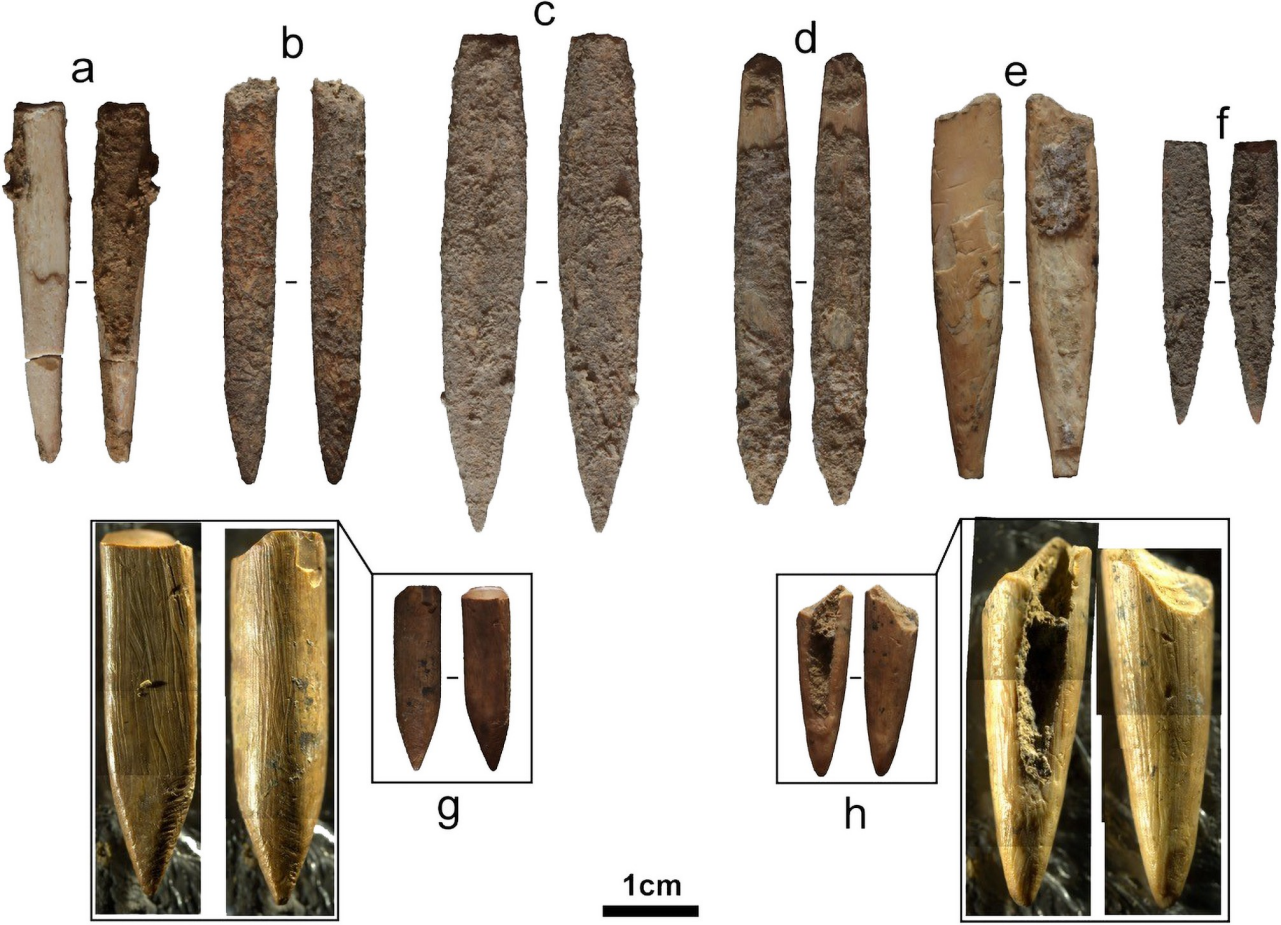
Osseous points. Pointed osseous artefacts from Leang Pajae and Leang Bulu’ Sipong 1 made on suid teeth.

Despite ‘bone points’ (Fig 19) being cited as characteristic of the Toalean culture of South Sulawesi [e.g. 2, 17], these artefacts have not yet been described in any great detail [but see 56, 150]. As a Toalean tool type, the technology is described here and associated terminology clarified. Initially, finds were described as “*pfeilspitzen aus knochen geschnitzt…* [and] *unterkeiferzähnen”* (“arrowheads carved of bone and tooth”) by the Sarasins [23 pl. 3, Figs 39–44], or “bone points (single and double pointed)” and “bone points of the Muduk type” by van Heekeren [2 p. 110–112]. *Muduk* is the Murundian (an Australian Aboriginal community in southeast Australia) word for ‘bone’ or ‘fishing-bone’ [151] and refers to small bipoints predominantly made on macropod bone. These are thought to have functioned as tips or barbs for fishing spears utilised along the coast and rivers of southeast Australia [151]. This term, brought over by the Australian archaeologist Frederick McCarthy who excavated with the Dutch in pre-World War Two South Sulawesi [7, 16], brings with it the implication that this tool form may be associated with fishing, although little evidence for their use as such a tool is yet found in the Sulawesi context. As with the label pirri point, use of the Australian term *muduk* for a Sulawesi tool-type is inappropriate, and, in our view, should be avoided in favour of descriptive terminology in line with global nomenclature [such as 62]. The label ‘bone’ point is also misleading, as a large portion are made on animal teeth (see below). Unlike Maros points, osseous points were not confined to the south arm of Sulawesi and have been recovered in sites throughout Sulawesi [e.g. 90, 150, 152, 153].

Here, 22 artefacts recovered from Leang Bulu’ Sipong 1 and Leang Pajae form the basis of the typology outlined, though data from artefacts described from Ulu Leang 1 and Leang Burung 1 by Olsen and Glover [56], and the Botocani karts area by Fakhri [154] was also utilised. Identification of osseous material relies on observation of physical attributes under low magnification and comparing these attributes to known faunal species.

As observed by Olsen and Glover [56 p. 285], “the paucity of worked bone in an otherwise rich faunal assemblage suggests that there was little dependence on bone as a raw material and that people may have relied more on hardwoods and bamboo”. As at Ulu Leang 1 and Leang Burung 1, the recovered osseous tools are very few and small in their overall dimensions. They are also not restricted to a particular raw material, with examples made on suid tooth, mammal cortical bone, and a possible example of avian bone, though tooth appears to be the material of choice (*n* = 20, 91%). The preference for suid tooth to manufacture points was previously noted in Aplin et al.’s [150] examination of similarly-aged osseous pointed tools from southeast Sulawesi.

Artefacts from Leang Bulu’ Sipong 1 and Leang Pajae (as well as those from Ulu Leang 1 and Leang Burung 1), represent small (c.50 mm to 11 mm long) points which appear to include both uni- and bipointed forms (Table 3). The coarse nature of preparation in some cases makes it difficult to establish if an item is a broken (in use) bipoint or a unipoint in a relatively complete state. All artefacts from Leang Bulu’ Sipong 1 and Leang Pajae appear to be projectile tips (which may include fishing spears or gorges), owing to their size and the observed damage to the extremities (crushing, chipping, snap fractures, splinter fractures, bevel fractures). The damage is not consistent with other tool uses such as plant or leather working [’awls’ in 56], though we cannot completely rule out multiple uses including use as ‘nosebones’, which, in the Australian context, can appear like the larger bipoints shown here. Sediments adhering to much of the surfaces of these Toalean artefacts obscures the use wear or residue traces (such as ochre) which would allow for further elucidation of their specific uses [155].

**Table 3:** Metrics for the osseous points of Leang Bulu’ Sipong 1 and Leang Pajae.

Site	Material	*n*	Length (mm)			Distal tip width (mm)	Midsection width (mm)	
			Range	Mean	SD^a^	Mean	Mean	SD^a^
Leang Bulu’ Sipong 1	animal bone	2	20.43–11.14	15.79	4.65	2.45	3.66	0.47
	Suidae tooth	14	46.02–15.18	26.55	9.12	1.97	4.59	0.89
Leang Pajae	Suidae tooth	6	50.23–16.35	32.99	12.24	2.18	4.07	1.97

^a^ Standard deviation.

Shaping of the points was undertaken by grinding, as indicated by the numerous sub-parallel striations visible on the bone/tooth surfaces not covered by sediment. These traces show that while the tool-maker took advantage of the tooth root to create a pointed tool, the shape was also accentuated by grinding (see Fig 19g), frequently revealing the pulp cavity (e.g., Fig 19h). Apart from being approximately the same length and width, further standardisation in form is missing–the points vary greatly in their symmetry and proportions (see examples in Fig 19). Further archaeological and experimental research is necessary to clarify the design and purpose of Toalean bone points, although it is clear that the use of suid tooth to create small pointed tools is a key aspect of osseous point technology in Sulawesi.

## Conclusion

The Toalean archaeology of South Sulawesi is central to several long-standing debates and narratives, including post-Pleistocene adaptations, early Holocene movements of people through and into ISEA and Australia, early maritime trade, and interactions between local hunter-gatherer populations and expanding Austronesian populations and spheres of influence. However, interpreting the nature and extent of the Toalean phenomenon–and its potential wider regional influence–has been hampered by inconsistent and contradictory classifications of the primary data used to define the Toalean: the stone and bone tool assemblages. Here we have proposed a more systematic framework for classifying this primary data to aid in more nuanced interpretations of the growing body of Toalean archaeological data.

Specifically, we suggest a primary division of retouched tools into 1) those shaped using the anvil-supported backing technique to produce microlith segments, and 2) those shaped using pressure flaking and/or freehand percussion. Although stylistic variability appears to occur in backed artefacts, we suggest that rather than characterising variability by creating typological categories it is better tracked through morphometric analyses that attempt to define discrete variations using empirical data. Different microlith styles may eventually emerge from this, but it is premature to assert those styles on present evidence. We also propose that retouched Maros points–the hallmark of the Toalean–in fact be divided into four subclasses: classic Maros Points, Mallinrung Points, Lompoa Points, and Pangkep Points. The relationship of these classes to each other–for instance, whether they reflect different stages of reduction of a single point category–can be explored in future analyses. We hope that divisions are useful, however, for assessing whether variations occur regionally. We have also characterised the essential characteristics of the Toalean osseous points, and suggest that further refinement of this artefact class can be approached through analysis, in particular, of the type of bone and teeth used to manufacture them.

We also propose that a unifying framework for the various stone tool types can be developed through reduction sequence analysis, combined with experimental archaeology focussed on making and using these stone tools. Our assessment suggests that the backed microlith technology is based on flakes rather than blades. Bipolar core reduction techniques are particularly important, as this serves as a technological connection to the use of the technique in earlier Pleistocene technologies in the region. Further, variations in reduction sequences can highlight technological differences between sites and regions that may not be apparent from the final tools themselves, as seen, for instance, in the technical differences in point manufacture between the Toalean assemblages and contemporary points from Japan, Java, and Australia.

Our analysis of the reduction process helps clarifies the place of Maros points in Toalean lithic technology. It appears that backed ‘tranchet point’ microliths are not early stages of Maros point manufacture, as has previously been suggested [85]. Backed tranchet points, as described by Glover and Presland [85], result from a reduction process that involved extensive modification through backing of both the proximal and distal ends of the flake blank, as opposed to minimal pressure-flaking work at the margins and proximal end, as we see on Maros points. It has also been suggested that Mallinrung points may be Maros points made by novices, and the base was too difficult to manufacture for less-confident knappers [20 p. 95]; however, in our experience, creating the fine margin denticulations is more challenging than the basal retouch (pers. obs. YLP).

In the search for cultural origins and connections, some prehistorians have compared the Maros points of South Sulawesi to other stone points in Australia and Southeast Asia. Within Indonesia, for example, Toalean points have been likened to the hollow-based points in assemblages from the Sampung industry, found in the Ponorogo and Pacitan regencies of Central and East Java [e.g. 2, 4, 16, 144, 156, 157]. Similar hollow-based bifacial points belong to several phases of the Jōmon period of Japan [2, 158, 159], and according to van Heekeren [16], may also have occurred in the Philippines and Korea. These apparent similarities have been interpreted as reflecting direct contact or long-distance cultural diffusion between the populations inhabiting these areas and the Toaleans of South Sulawesi [e.g. 2, 4]. However, the stone points found in Java and Japan lack the delicate edge denticulations produced in South Sulawesi, and these points were invasively flaked through very different pressure-flaking gestures. It is possible that serrated stone points were also produced in China [160, not illustrated] and Korea [161, in 162], but this is difficult to verify. Authors have also drawn comparisons with various Australian points, including the diverse forms of Kimberley points, pirri points, and Bondi/Woakwine points [5–7, 24]. Kimberley points, in particular, are often serrated or denticulated [60]. However, none of the Australian points have an indented base, they are usually invasively flaked by pressure flaking–sometimes after a percussion thinning stage–and they tend to have a comparatively thick cross-section [60, 145]. These technological differences and the reduction processes that created them need to be considered when making typological comparisons, as morphology alone may reflect convergence [84, 132].

With the discovery of a presently unknown early hominin presence [36], the oldest known surviving figurative rock art in the world [38], and unforeseen evidence for interaction between hunter-gatherers and migrating farmers, Sulawesi has emerged as one of the key regions internationally for understanding human cultural adaptations and movements in the far-distant and recent past. We hope that that our framework will assist in providing a robust basis for evaluating some of this evidence for future local and international archaeological research agendas. It is anticipated that this standardised model and type definitions of Toalean artefacts may provide a common system for future in-depth archaeological research in this important region.

## Supporting information

S1 Text Abstrak Bahasa Indonesia. Alternative Language Abstract (Indonesian).
(PDF)

S1 Table Metric data for sawlettes, Toalean points, and osseous points. Attribute definitions are given in the text.
(PDF)
