# Supplementary material for: A standardised classification scheme for the Mid-Holocene Toalean artefacts of South Sulawesi, Indonesia
Source: PLoS One. 2021 May 26;16(5):e0251138. doi: 10.1371/journal.pone.0251138 (PMC8153489; doi:10.1371/journal.pone.0251138)
Supplement: S1 Text — Alternative Language Abstract (Indonesian). (PDF) [file pone.0251138.s001.pdf]

S1 Text. Abstrak Bahasa Indonesia. Alternative Language Abstract (Indonesian).

Arkeologi Sulawesi penting untuk mengembangkan pemahaman mengenai persebaran manusia dan hunian di tengah-tengah pulau Asia Tenggara. Setelah lebih dari satu abad pekerjaan arkeologi, berbagai gelombang pendudukan manusia di wilayah barat daya Sulawesi telah diidentifikasi, yang paling terdokumentasi dengan baik adalah dari periode teknologi 'Toalean' Holosen Tengah hingga Akhir. Model arkeologi untuk periode ini menggambarkan populasi dengan identitas budaya yang kuat, terbagi menjadi kelompok yang berkembang di bagian pesisir sekitar Maros-Pangkep serta penghuni hutan dataran tinggi yang tersebar dan berburu satwa liar endemik dengan teknologi busur-panah. Diusulkan bahwa Toalean mampu menyeberangi air, dan bahkan melakukan pertukaran budaya dengan Australia utara, Jawa, dan Jepang. Gambaran ini secara eksklusif bergantung pada pemahaman terkini tentang teknologi khas batu Toalean dan artefak tulang, tetapi model ini pada gilirannya dan didasarkan pada filosofi abad ke-19 dan ke-20 yang sudah ketinggalan zaman. Definisi tipe artefak Toaean diterapkan secara tidak konsisten dan tidak sistematis, dan urutan pembuatannya secara historis kurang dipahami. Untuk mengatasi masalah pada sumber daya fundamental ini, makalah ini menyajikan tipologi yang diperjelas dari artefak-artefak Toalean di Sulawesi Selatan, dan menjelaskan aspek teknis produksi artefaknya. Tipologi ini menyediakan alat untuk menstandarisasi penelitian dan akan menfasilitasi penilaian yang lebih bermakna dari himpunan budaya material dan penilaian perubahan spasial dan temporal yang lebih dapat diandalkan.
