# Supplementary material for: A standardised classification scheme for the Mid-Holocene Toalean artefacts of South Sulawesi, Indonesia
Source: PLoS One. 2021 May 26;16(5):e0251138. doi: 10.1371/journal.pone.0251138 (PMC8153489; doi:10.1371/journal.pone.0251138)
Supplement: S1 Table — Attribute definitions are given in the text. (PDF) [file pone.0251138.s002.pdf]

S1 Table. Metric data for sawlettes, Toalean points, and osseous points. Attribute definitions are given in the text.

Table A. Dataset for sawlettes.

#	Site	Situation	Depth (cm)	Material	Length (mm)	Width (mm)	Thickness (mm)	Weight (g)	Sample denticulation-scar platform (mm)	Sample denticulation depth (mm)	Sample denticulation spacing (mm)	No. of denticulations	Condition
1	Leang Bulu' Sipong 1	T9S1	30-40	chert	17.45 (broken)	3.76	1.71	0.12	0.65	0.57	2.10	7	
2	Leang Bulu' Sipong 1	T9S1	30-40	chert	17.48	5.67	2.83	0.2	0.90	0.80	2.18	7	
3	Leang Bulu' Sipong 1	T9U1	20-30	chert	10.27 (broken)	4.95	1.97	0.12	0.93	1.00	1.98	4	heat damage
4	Leang Bulu' Sipong 1	T9S1	10-20	chert	17.91 (broken)	3.58	1.87	0.17	0.58	1.00	2.19	10	
5	Leang Bulu' Sipong 1	T9S1	10-20	chert	15.67 (broken)	4.49	2.22	0.19	unid.	unid.	unid.	unid.	heat damage
6	Leang Bulu' Sipong 1	T9S1	30-40	chert	13.96 (broken)	4.02	2.01	0.11	0.49	1.59	2.83	3	
7	Leang Bulu' Sipong 1	T9S1	0-10	chert	10.86	3.33	1.52	0.07	0.52	0.68	1.64	6	
8	Leang Bulu' Sipong 1	T9S1	10-20	chert	14.4	4.79	1.83	0.15	0.91	0.70	2.03	6	
9	Leang Bulu' Sipong 1	T9S1	20-30	chert	15.64 (broken)	3.99	1.36	0.13	0.61	0.79	1.98	8	
10	Leang Bulu' Sipong 1	T9S1	50-60	chert	10.93 (broken)	3.3	1.5	0.08	0.76	0.53	1.28	6	
11	Leang Bulu' Sipong 1	T9S1	30-40	chert	14.49 (broken)	4.34 (broken)	1.27	0.09	0.66	0.63	1.55	8	heat damage
12	Leang Bulu' Sipong 1	T9S1	20-30	chert	14.83 (broken)	3.54	1.68	0.12	0.79	0.63	2.17	6	
13	Leang Jarie	S3B1	150-160	chert	17.53	3.24	1.74	0.14	unid.	unid.	unid.	8	
14	Leang Jarie	S4T1	40-50	chert	14.89	3.77	1.2	0.13	unid.	unid.	unid.	7	

Table B. Dataset for Toalean points.

#	Site	Situation	Point type	Material	Length (mm)	Width (mm)	Thickness (mm)	Weight (g)	Max. retouching scar length (mm)	Max. retouching scar width (mm)	Sample width of denticulation-scar platform (mm)	Sample denticulation length/depth (mm)	Sample denticulation spacing (mm)	Flake thickness at base of denticulation (mm)	No. of denticulations	width of basal notch (mm)	depth of basal notch (mm)	Condition	Bipolar
1	Lambatorang	surface find	classic Maros point	chert	16.67 (broken)	17.18	3.34	0.68	2.86	3.51	0.96					8.67	4.21	heat damage	
2	Lambatorang	surface find	Lompoa	chert	26.45	12.63	3.26	0.89	2.47	3.12						8.11	2.65		
3	Lambatorang	surface find	unid.	chert	20.35	10.90	2.82	0.49	1.69	2.24	1.10					5.79	2.7		
4	Lambatorang	surface find	classic Maros point, incomplete	chert	18.77	11.72	4.02	0.76	3.89	4.98	1.09					8.6	1.22		
5	Lambatorang	surface find	classic Maros point	chert	17.95 (broken)	13.51	3.37	0.66	4.29	3.90	1.10					8.14	4.38		
6	Lambatorang	surface find	Lompoa	chert	27.86	13.46	3.44	1.17	10.23	11.21	0.68					8.66	5.1		
7	Lambatorang	surface find	unid.	chert	11.47 (broken)	7.90 (broken)	1.48	0.10	1.65	1.60	0.74								
8	Leang Bulu' Sipong 1	T9S1	unid.	chert	12.21 (broken)	9.03	1.91	0.21	0.87	1.52	0.50	0.89	2.32	0.55	5				
9	Leang Bulu' Sipong 1	T9S1	unid.	chert	21.30 (broken)	10.10	1.67	0.41	0.83	2.06	0.95	1.32	1.93	0.57	7				

10	Leang Bulu' Sipong 1	T9S1	classic Maros point	chert	19.16	8.04	2.33	0.33	1.97	2.96	0.76	0.66	1.61	0.58	9	4.95	3.21		
11	Leang Bulu' Sipong 1	T9S1	unid.	chert	16.79 (broken)	11.80	2.64	0.44	1.24	1.50	0.62	1.14	2.18	0.6	4			heat damage	
12	Leang Bulu' Sipong 1	T9S1	classic Maros point	chert	20.78 (broken)	9.20	2.07	0.32	1.53	2.35	0.57	0.61	1.77	0.68	14				
13	Leang Bulu' Sipong 1	T9S1	classic Maros point	chert	23.43 (broken)	16.22	3.21	0.65	1.37	2.69	0.75	1.70	3.37	0.68	7				
14	Leang Bulu' Sipong 1	T9S1	classic Maros point	chert	7.79 (broken)	7.77 (broken)	1.52	0.12	0.66	0.86	0.73	0.60	0.92	0.68	6				
15	Leang Bulu' Sipong 1	T9S1	unid.	chert	18.45 (broken)	19.18	3.25	1.14	0.96	2.03	0.50	0.70	1.86	0.72	12				
16	Leang Bulu' Sipong 1	T9S1	Mallinrung point	chert	22.66	11.98	2.40 (broken)	0.57	0.73	1.83	0.64	1.78	2.70	0.75				heat damage	
17	Leang Bulu' Sipong 1	T9S1	classic Maros point	chert	21.01 (broken)	10.18	1.81	0.25	1.10	2.64	0.46	1.66	2.44	0.75	15				
18	Leang Bulu' Sipong 1	T9S1	unid.	chert	16.75 (broken)	14.93 (broken)	2.94	0.70	1.36	1.59	0.29	0.58	1.73	0.75	7	8.67	1.24		
19	Leang Bulu' Sipong 1	T9S1	unid.	chert	22.44 (broken)	11.43	3.29	0.86	1.44	2.53	1.18	2.35	4.24	0.75	4			heat damage	
20	Leang Bulu' Sipong 1	T9S1	unid.	chert	19.89 (broken)	14.13	2.04	0.67	1.09	2.19	0.76	0.88	1.80	0.76	10			heat damage	
21	Leang Bulu' Sipong 1	T9S1	unid.	chert	20.17 (broken)	5.86	2.10	0.18	1.20	2.40	0.71	0.95	2.56	0.76	12				
22	Leang Bulu' Sipong 1	T9S1	classic Maros point	chert	21.50	12.33	2.02 (broken)	0.44	1.94	1.66	0.81	1.18	1.89	0.77	12			heat damage	
23	Leang Bulu' Sipong 1	T9S1	Mallinrung point	chert	37.06	14.57	4.33	1.56	0.87	3.20	0.98	1.61	3.21	0.79	17				
24	Leang Bulu' Sipong 1	T9S1	classic Maros point	chert	11.04 (broken)	12.34	2.09	0.22	1.14	2.02	0.61	1.20		0.8	2	7.2	4.16		
25	Leang Bulu' Sipong 1	T9S1	unid.	chert	21.92 (broken)	16.98	3.34	0.70	1.06	1.62	0.95	1.51	3.04	0.83	9			heat damage	

26	Leang Bulu' Sipong 1	T9S1	unid.	chert	14.57	11.69	1.90	0.33	1.34	1.95	0.55	1.51	2.00	0.83	5	6.03, 5.42	2.61, - 2.56		
27	Leang Bulu' Sipong 1	T9S1	classic Maros point	chert	19.47	8.74	1.93	0.29	1.63	1.98	1.05	0.86	2.23	0.86	6	4.72	2.64		
28	Leang Bulu' Sipong 1	T9S1	classic Maros point	chert	23.85	14.75 (broken)	2.84	0.83	1.90	2.88	0.89	1.26	2.32	0.86	7	7.79	3.21		
29	Leang Bulu' Sipong 1	T9S1	classic Maros point	chert	26.36	13.36	3.60	0.88	2.53	2.52	0.35	1.51	2.41	0.87	23	10.5	4.09		
30	Leang Bulu' Sipong 1	T9S1	classic Maros point	chert	20.38	14.13	3.35	0.90	1.64	2.45	0.75	0.72	1.94	0.88	16	7.46	3.74		
31	Leang Bulu' Sipong 1	T9S1	classic Maros point	chert	26.28	14.30	4.43	1.21	2.87	3.09	0.69	0.55	1.41	0.89		9.66	4.49		
32	Leang Bulu' Sipong 1	T9S1	unid.	chert	20.86 (broken)	12.65	2.98	0.53	1.77	2.57	0.53	1.56	3.31	0.9	4				
33	Leang Bulu' Sipong 1	T9S1	classic Maros point	chert	17.09	11.16	2.44	0.36	2.06	2.80	1.04	1.90	2.66	0.91	9	6.08	3.01		
34	Leang Bulu' Sipong 1	T9S1	classic Maros point	chert	21.61	11.33	1.99	0.33	1.62	1.63	0.73	2.07	2.78	0.91	13	5.34	3.4		
35	Leang Bulu' Sipong 1	T9S1	classic Maros point	chert	12.43 (broken)	14.76	3.69	0.74	3.08	4.33	0.47	1.05	3.27	0.92	3	11.58	1.95		
36	Leang Bulu' Sipong 1	T9S1	classic Maros point	chert	17.49 (broken)	10.51 (broken)	2.52	0.30	2.52	2.66	0.47	1.58	2.32	0.93	7				
37	Leang Bulu' Sipong 1	T9S1	classic Maros point, incomplete	chert	22.28 (broken)	11.44	2.29	0.52	1.55	3.42	1.02	1.96	2.55	0.94	6	7.35	4.26		

38	Leang Bulu' Sipong 1	T9S1	classic Maros point, incomplete	chert	18.05 (broken)	12.82	2.80	0.63	2.38	3.23	0.54	1.44	2.87	0.94	1	6.2	5.05	edge gloss	
39	Leang Bulu' Sipong 1	T9S1	unid.	chert	20.25 (broken)	15.84	3.16	1.06	2.21	3.63	0.50	2.51	3.36	0.94	6	11.36	5.98		
40	Leang Bulu' Sipong 1	T9S1	unid.	chert	16.35 (broken)	11.82 (broken)	2.56	0.34	2.45	3.24	0.75	1.80	2.86	0.94	5				
41	Leang Bulu' Sipong 1	T9S1	classic Maros point, incomplete	chert	25.98	14.38	3.27	1.28	3.09	5.04	0.77	1.90	2.94	0.95	3	8.25	3.24		
42	Leang Bulu' Sipong 1	T9S1	classic Maros point, incomplete	chert	24.11 (broken)	13.64 (broken)	2.74	0.82	1.97	1.75	0.54	1.81	2.57	0.95	4				
43	Leang Bulu' Sipong 1	T9S1	classic Maros point	chert	11.66 (broken)	11.38	1.91	0.23	1.61	2.93	0.56	2.16	2.38	0.96	6	5.12	3.43	heat damage	
44	Leang Bulu' Sipong 1	T9S1	classic Maros point	chert	26.28	13.58	3.32	0.85	2.47	3.03	0.80	1.64	2.59	0.97	23	10.14	3.2		
45	Leang Bulu' Sipong 1	T9S1	classic Maros point	chert	31.32 (broken)	12.69	2.68	0.89	2.36	3.22	0.75	1.88	3.75	0.97	11				
46	Leang Bulu' Sipong 1	T9S1	unid.	chert	22.59 (broken)	11.50 (broken)	2.52	0.72	2.22	2.19	0.62	1.20	1.85	0.97	6			heat damage	
47	Leang Bulu' Sipong 1	T9S1	unid.	chert	25.65 (broken)	11.11	2.72	0.50	1.47	2.35	0.88	2.12	2.73	0.97	8				
48	Leang Bulu' Sipong 1		unid.	chert	10.45 (broken)	6.58 (broken)	2.13	0.18	1.76	3.32	1.03	1.14	1.97	1	5				
49	Leang Bulu' Sipong 1	T9S1	classic Maros point, incomplete	chert	19.09 (broken)	15.91	2.12	0.67	1.68	2.75	0.68	1.64	3.06	1.01	4	8.43	6		
50	Leang Bulu' Sipong 1	T9S1	unid.	chert	16.30 (broken)	11.82	2.49	0.70	1.73	3.11	0.53	0.98	2.58	1.01	3				
51	Leang Bulu' Sipong 1	T9S1	unid.	chert	22.65 (broken)	12.98 (broken)	1.75	0.43	1.63	2.31	0.46	1.28	2.55	1.03	9				

52	Leang Bulu' Sipong 1	T9S1	classic Maros point	chert	25.74 (broken)	11.62	2.43	0.52	1.62	2.56	0.55	1.76	2.21	1.04	19				
53	Leang Bulu' Sipong 1	T9S1	classic Maros point	chert	22.31	14.18	2.44	0.56	2.66	2.84	0.80	1.85	2.21	1.04	14	8.64	4.62		
54	Leang Bulu' Sipong 1	T9S1	classic Maros point	chert	16.30 (broken)	12.26	3.14	0.49	?	?	?	0.58		1.04	2		4.4		
55	Leang Bulu' Sipong 1	T9S1	unid.	chert	23.59 (broken)	12.47	2.19 (broken)	0.46	1.88	2.66	1.14	1.58	3.50	1.04	3			heat damage	
56	Leang Bulu' Sipong 1	T9S1	classic Maros point	chert	11.08 (broken)	14.13	2.91	0.44	1.36	1.82	0.55	0.86	2.53	1.06	5	6.21	2.92		
57	Leang Bulu' Sipong 1	T9S1	classic Maros point	chert	24.14	11.36	2.06	0.56	1.95	3.28	0.78	1.78	2.91	1.07	11	6.37	4.76		
58	Leang Bulu' Sipong 1	T9S1	classic Maros point	chert	13.16 (broken)	10.53 (broken)	2.04	0.31	1.88	3.22	0.77	1.49	2.61	1.07	4				
59	Leang Bulu' Sipong 1	T9S1	unid.	chalcedony	19.15 (broken)	15.82	2.41	0.79	1.68	2.76	1.06	1.85	5.06	1.07	2				
60	Leang Bulu' Sipong 1	T9S1	classic Maros point	chert	24.59	14.46	2.28	0.85	1.93	3.56	0.60	2.43	3.52	1.08	10	10.71	5.53		
61	Leang Bulu' Sipong 1	T9S1	unid.	chert	16.21 (broken)	12.43 (broken)	3.40	0.63	1.90	2.51	0.89	1.30	2.31	1.09				heat damage	
62	Leang Bulu' Sipong 1	T9S1	classic Maros point	chert	24.81 (broken)	13.85	1.96	0.60	2.20	2.59	0.85	2.91	3.70	1.11	13				
63	Leang Bulu' Sipong 1	T9S1	classic Maros point, incomplete	chert	16.03 (broken)	10.93 (broken)	1.99	0.38	2.39	2.02	0.68	1.80	3.16	1.12	2	6.32	2.95		
64	Leang Bulu' Sipong 1	T9S1	classic Maros point, incomplete	chert	17.20 (broken)	11.17	3.53	0.58	1.72	2.71	0.62	1.14	2.74	1.13					

65	Leang Bulu'<br>Sipong 1	T9S1	classic<br>Maros<br>point	chert	8.79<br>(broken)	12.61	2.27	0.28	2.79	2.92	0.97	1.94	2.88	1.13	4				
66	Leang Bulu'<br>Sipong 1	T9S1	classic<br>Maros<br>point	chert	18.80<br>(broken)	18.92	3.86	1.43	2.52	4.91	0.57	1.38	2.65	1.14	3	14.09	4.63		
67	Leang Bulu'<br>Sipong 1	T9S1	classic<br>Maros<br>point	chert	23.57	13.77	2.76	0.51	2.70	2.76	0.76	1.56	3.43	1.15	10	7.43	3.99		
68	Leang Bulu'<br>Sipong 1	T9S1	classic<br>Maros<br>point	chert	22.71<br>(broken)	9.63	3.00	0.47	3.01	3.86	0.85	1.50	2.95	1.15	14				
69	Leang Bulu'<br>Sipong 1	T9S1	classic<br>Maros<br>point	chert	12.74<br>(broken)	11.13	2.33	0.36	2.04	3.55	0.97	1.44	4.06	1.15	2				
70	Leang Bulu'<br>Sipong 1	T9S1	classic<br>Maros<br>point,<br>incomplete	chert	31.04	17.04	3.33	1.23	2.60	3.22	0.64	2.87	3.42	1.17	5				
71	Leang Bulu'<br>Sipong 1	T9S1	classic<br>Maros<br>point	chert	22.19	11.07	2.17	0.48	2.37	2.60	0.75	1.34	3.14	1.17	12	7.23	4.36		
72	Leang Bulu'<br>Sipong 1	T9S1	classic<br>Maros<br>point	chert	23.79	11.23	2.47	0.58	1.57	3.29	0.59	1.44	2.98	1.18	15	6.69	2.42		
73	Leang Bulu'<br>Sipong 1	T9S1	unid.	chert	22.08<br>(broken)	15.25<br>(broken)	2.21	0.53	1.56	2.52	0.56	2.21	3.74	1.19	9				
74	Leang Bulu'<br>Sipong 1	T9S1	classic<br>Maros<br>point	chert	15.41<br>(broken)	16.67	2.64	0.57	2.24	1.36	0.57	2.41	3.49	1.2	6	9.52	4.6	heat<br>damage	
75	Leang Bulu'<br>Sipong 1	T9S1	classic<br>Maros<br>point	chert	19.62<br>(broken)	16.87	4.21	1.09	4.21	4.27	1.09	1.58	2.93	1.21	10	7.42	4.93		
76	Leang Bulu'<br>Sipong 1	T9S1	classic<br>Maros<br>point	chert	18.81<br>(broken)	16.71	3.31	0.82	5.35	2.06	0.96	1.57	3.36	1.21	8	7.57	4.99		

77	Leang Bulu' Sipong 1	T9S1	classic Maros point	chert	24.19	11.38	2.12	0.55	1.72	3.51	0.77	1.86	3.12	1.22	10	7.47	5		
78	Leang Bulu' Sipong 1	T9S1	classic Maros point, incomplete	chert	18.32 (broken)	14.43	3.90	0.90	2.23	3.06	0.83	1.53	3.07	1.23	5	9.02	3.04	heat damage	
79	Leang Bulu' Sipong 1	T9S1	classic Maros point	chert	18.59 (broken)	14.36 (broken)	1.86 (broken)	0.37	1.57	2.11	1.53			1.27	1			heat damage	
80	Leang Bulu' Sipong 1	T9S1	classic Maros point	chert	29.86	11.86	3.30	0.77	2.77	3.77	0.64	2.70	3.00	1.28	20				
81	Leang Bulu' Sipong 1	T9S1	classic Maros point	chert	29.85 (broken)	12.73	3.74	1.02	4.11	3.79	0.49	2.70	4.06	1.28	10				
82	Leang Bulu' Sipong 1	T9S1	classic Maros point, incomplete	chert	20.40	10.89	2.94	0.51	2.08	3.30	0.84	0.81	2.11	1.29	6	6.83	2.89		
83	Leang Bulu' Sipong 1	T9S1	unid.	chert	38.05 (broken)	17.12	4.20	2.28	2.37	3.33	0.77	1.18	2.03	1.29	5				
84	Leang Bulu' Sipong 1	T9S1	classic Maros point, incomplete	chert	24.26 (broken)	12.05	2.89	0.65	2.70	2.98	0.56	1.95	3.28	1.3	8	6.11	4.25		
85	Leang Bulu' Sipong 1	T9S1	Mallinrung point	chalcedony	26.67	9.69	3.28	0.60	2.52	4.15	0.66	1.69	2.96	1.32	11				
86	Leang Bulu' Sipong 1	T9S1	classic Maros point	chert	17.72 (broken)	13.57	2.80	0.58	2.49	3.29	0.42	2.64	4.00	1.32	7	9.59	3.9		
87	Leang Bulu' Sipong 1	T9S1	classic Maros point, incomplete	chert	18.44 (broken)	14.48	3.36	0.87	3.67	3.89	0.88	1.56	3.40	1.34	3				
88	Leang Bulu' Sipong 1	T9S1	unid.	chert	24.71 (broken)	13.64	3.28	0.87	2.50	3.39	0.77	2.29	2.93	1.36	3				
89	Leang Bulu' Sipong 1	T9S1	Mallinrung point	chert	26.17 (broken)	12.17	4.02	1.24	2.30	4.02	0.53	1.71	2.76	1.37	8			heat damage	

90	Leang Bulu' Sipong 1	T9S1	classic Maros point	chert	22.95	15.77	3.66	1.02	2.04	2.70	0.42	1.68	2.72	1.38	11	7.33	2.7		
91	Leang Bulu' Sipong 1	T9S1	classic Maros point	chert	28.77	13.43	4.96 (broken)	1.02	3.54	2.41	0.75	1.44	3.05	1.39	17			heat damage	
92	Leang Bulu' Sipong 1	T9S1	classic Maros point	chert	24.50	12.20	4.29	0.91	3.13	3.76	0.70	1.97	2.75	1.41	9	8.84	3.98		
93	Leang Bulu' Sipong 1	T9S1	classic Maros point	chert	22.16 (broken)	12.82	3.16	0.61	2.39	3.41	0.74	2.03	4.18	1.42	8				
94	Leang Bulu' Sipong 1	T9S1	classic Maros point	chert	27.43 (broken)	15.84	4.32	1.13	4.48	5.00	0.69	3.02	4.87	1.43	8				
95	Leang Bulu' Sipong 1	T9S1	classic Maros point	chert	18.40	12.50	3.43	0.50	2.87	2.40	0.80	2.42	3.72	1.47	6	8.46	3.98		
96	Leang Bulu' Sipong 1	T9S1	classic Maros point	chert	29.50	17.41	3.40	1.09	2.09	4.34	0.78	2.10	3.14	1.51	13	12.09	4.52		
97	Leang Bulu' Sipong 1	T9S1	classic Maros point	chert	25.25	14.53	3.88	0.90	2.95	5.12	0.65	2.22	3.28	1.51	9	8.62	5.29		
98	Leang Bulu' Sipong 1	T9S1	classic Maros point	chert	23.64	13.07	2.92	0.72	2.71	3.25	0.40	2.45	3.09	1.56	13	7.19	4.95		
99	Leang Bulu' Sipong 1	T9S1	classic Maros point	chert	24.80 (broken)	20.14	4.94	1.83	6.42	6.35	0.65	3.15	3.37	1.57	12	16.15	5.2		
100	Leang Bulu' Sipong 1	T9S1	unid.	chert	10.75 (broken)	9.54 (broken)	2.97	0.27	2.79	3.84	0.50	2.22	3.47	1.6	2				
101	Leang Bulu' Sipong 1	T9S1	classic Maros point	chalcedony	18.81 (broken)	15.76	2.83	0.81	2.71	4.04	0.98	2.32	3.22	1.61	8	7.33	3.58		
102	Leang Bulu' Sipong 1	T9S1	classic Maros point	chert	21.47 (broken)	12.30 (broken)	3.63	1.19	2.69	4.18	0.62	1.94	2.91	1.63	6				

103	Leang Bulu' Sipong 1	T9S1	classic Maros point	chert	10.02 (broken)	6.90 (broken)	2.20 (broken)	0.13	1.96	3.23	0.39	1.31	2.76	1.65	3			heat damage	
104	Leang Bulu' Sipong 1	T9S1	classic Maros point	chert	14.86 (broken)	13.82	2.71	0.55	1.73	1.59	0.84		4.84	1.68	3	8.81	3.36		
105	Leang Bulu' Sipong 1	T9S1	classic Maros point	chert	27.59 (broken)	14.26	3.96	1.18	4.48	4.37	0.99	2.12	3.86	1.68	13				
106	Leang Bulu' Sipong 1	T9S1	classic Maros point, incomplete	chert	32.61	16.42	3.77	1.69	2.93	4.30	0.70	1.95	3.97	1.72	13	9.93	4.8		
107	Leang Bulu' Sipong 1	T9S1	classic Maros point	chalcedony	34.99	17.67	5.03	3.09	3.64	5.19	1.10	1.39	3.94	1.75	6				
108	Leang Bulu' Sipong 1	T9S1	classic Maros point	chert	22.07	14.90	2.67	0.72	2.38	4.55	0.68	2.63	4.36	1.79	6	6.66	3.52		
109	Leang Bulu' Sipong 1	T9S1	unid.	chert	41.60	19.31	4.68	3.31	4.04	5.79	0.60	1.35	3.13	1.84	9			usewear	
110	Leang Bulu' Sipong 1	T9S1	classic Maros point	chert	26.66	15.86	4.88	1.08	2.38	3.18	0.78	2.37	4.61	1.87	8	8.54	4		
111	Leang Bulu' Sipong 1	T9S1	unid.	chert	24.23 (broken)	22.26	3.95	1.27	2.69	4.41	1.18	2.54	3.72	2.02	4				
112	Leang Bulu' Sipong 1	T9S1	Lompoa	chalcedony	15.73 (broken)	17.50	2.21	0.61	1.53	1.50					0	6.34	5.3		
113	Leang Bulu' Sipong 1	T9S1	Lompoa	chert	29.56	14.68	3.51	1.18	2.70	2.88						9.88	4.3	edge gloss	
114	Leang Bulu' Sipong 1	T9S1	Lompoa	chert	22.34	13.67	3.37	0.77	2.95	3.69	0.51						5.52	heat damage	
115	Leang Bulu' Sipong 1	T9S1	classic Maros point	chert	23.99	9.92	4.00	0.72	3.05	4.02	0.69					9.34	2.89	heat damage	
116	Leang Bulu' Sipong 1	T9S1	classic Maros point, incomplete	chert	29.15 (broken)	10.33	3.67	1.17	2.12	3.86					1	6.12	3.05	heat damage	

117	Leang Bulu' Sipong 1	T9S1	Lompoa	chert	27.69	11.35	3.42	0.94	2.93	2.67						7.42	3.66		
118	Leang Bulu' Sipong 1	T9S1	Lompoa	chert	18.26	13.27	2.49	0.57	2.67	2.15	0.81				0	8.1	4.01		
119	Leang Bulu' Sipong 1	T9S1	Lompoa	chert	20.71 (broken)	15.43	2.81	0.72	1.70	1.26									
120	Leang Bulu' Sipong 1	T9S1	Lompoa	chert	22.04	13.79	3.47	0.86	3.72	2.84						8.74	3.84		
121	Leang Bulu' Sipong 1		classic Maros point, incomplete	chert	23.63 (broken)	12.21	3.29	1.00	3.34	4.64	0.65				2	7.97	5.61		
122	Leang Bulu' Sipong 1	T9S1	Lompoa	chert	23.02	12.23	2.93	0.69	1.89	3.39						7.94	4.19		
123	Leang Bulu' Sipong 1	T9S1	Lompoa	chert	22.37	16.57	3.61	1.15	2.99	2.99						11.03	5.69		
124	Leang Bulu' Sipong 1	T9S1	Lompoa	chert	33.49	17.40	5.19	2.45	2.44	1.66						7.56	3.54		
125	Leang Bulu' Sipong 1	T9S1	Lompoa	chert	31.01	18.33	4.46	2.23								10.83	3.9		
126	Leang Bulu' Sipong 1	T9S1	Pangkep	chert	24.24	17.30	4.62	1.47								13.89	3.11		
127	Leang Bulu' Sipong 1	T9S1	classic Maros point	chert	24.03	11.91	2.86	0.69	1.83	2.16	0.76	0.82	2.09		18	7.89	3.98		
128	Leang Bulu' Sipong 1	T9S1	Lompoa	chert	25.42	11.92	4.48	1.03	3.04	2.36						6.46	3.46	edge gloss	
129	Leang Bulu' Sipong 1	T9S1	Lompoa	chert	20.47	13.06	3.36	0.71	1.54	2.43	1.43					7.54	3.99		
130	Leang Bulu' Sipong 1	T9S1	Lompoa	chert	24.69 (broken)	13.92	3.40	0.96	1.42	1.47									
131	Leang Bulu' Sipong 1	T9S1	Lompoa	chert	21.43 (broken)	11.95	4.12	1.06	3.69	4.39						7.36	2.57		
132	Leang Bulu' Sipong 1	T9S1	Lompoa	chert	26.62	14.15	3.28	1.11	3.18	3.17									
133	Leang Bulu' Sipong 1	T9S1	Lompoa	chert	18.64	12.25	2.49	0.56											
134	Leang Bulu' Sipong 1	T9S1	Lompoa	chert	34.81	16.14	2.92	1.47	2.53	3.82						9.38	5.01		

135	Leang Bulu' Sipong 1	T9S1	Lompoa	chert	36.14	17.04	3.89	2.13	4.93	4.50	0.61				0	10.08	8.41		
136	Leang Bulu' Sipong 1	T9S1	Lompoa	chert	21.94 (broken)	14.78	3.21	0.78	3.59	2.51									
137	Leang Bulu' Sipong 1	T9S1	Lompoa	chert	26.98	14.70	3.82	1.37	2.24	4.07						11.46	5.63		
138	Leang Bulu' Sipong 1	T9S1	Lompoa	chert	22.59 (broken)	15.92	2.49	1.20	2.38	2.23						8.44	3.99		
139	Leang Bulu' Sipong 1	T9S1	Lompoa	chert	33.03	14.21	4.14	1.22	2.96	2.41						7.47	3.88		
140	Leang Bulu' Sipong 1	T9S1	unid.	chert	26.13	20.43	3.47	1.57			1.09					11.15	2.45	heat damage	
141	Leang Bulu' Sipong 1	T9S1	unid.	chert	26.17	20.32	3.46	1.59			0.68					7.85	2.43		
142	Leang Bulu' Sipong 1	T9S1	unid.	chert	15.96 (broken)	13.30	2.38	0.55								5.53	4.17		
143	Leang Bulu' Sipong 1	T9S1	unid.	chert	8.59 (broken)	11.69	1.98	0.26								5.25	2.4		
144	Leang Bulu' Sipong 1	T9S1	Lompoa	chalcedony	23.06	12.11	3.09	0.57	2.89	1.94					0				
145	Leang Bulu' Sipong 1	T9S1	Lompoa	chalcedony	20.02	12.71	2.58	0.86	2.77	2.10						7.99	5.51		
146	Leang Bulu' Sipong 1	T9S1	Pangkep	chert	31.29	14.02	5.30	2.32	3.01	2.85					0				
147	Leang Bulu' Sipong 1	T9S1	Lompoa	chert	31.11	15.02	4.20	1.65	4.11	5.14									
148	Leang Bulu' Sipong 1	T9S1	Lompoa	chert	10.29 (broken)	11.83	2.70	0.40	1.79	2.99						10.78	2.11		
149	Leang Bulu' Sipong 1	T9S1	classic Maros point, incomplete	chert	28.45 (broken)	15.87	4.20	1.57	1.78	2.62	1.20	1.27			3				
150	Leang Bulu' Sipong 1	T9S1	unid.	chert	21.08	17.74	3.60	1.04	1.11	3.12		1.21			1				
151	Leang Bulu' Sipong 1	T9S1	unid.	chert	20.07 (broken)	12.84	2.87	0.74	2.26	3.38									
152	Leang Bulu' Sipong 1	T9S1	unid.	chert	13.31 (broken)	14.53	2.74	0.58	2.62	2.66									

153	Leang Bulu' Sipong 1	T9S1	unid.	chert	27.91 (broken)	19.04	4.76	1.75	1.23	2.49	0.64								
154	Leang Lompoa	surface find	Lompoa	chert	24.80	12.44	3.24	0.88	3.02	2.48						9.51	3.89		
155	Leang Pajae	TP1	unid.	chert	22.00 (broken)	20.23	2.89	1.61	2.56	3.32	0.59	0.55	2.75	0.57				edge gloss	1
156	Leang Pajae	TP1	Pangkep	chert	25.49	17.63	4.64	1.20	2.47	4.77			2.51	0.59					
157	Leang Pajae	TP1	unid.	chert	20.50	14.56	3.93	1.02	1.84	3.06	0.60	0.83	2.59	0.77					
158	Leang Pajae	TP1	Pangkep	chert	31.98	13.11	2.88	1.17	3.10	3.69	0.49			0.77					
159	Leang Pajae	TP1	Mallinrung point	chert	19.65	13.49	4.15	0.63	2.76	4.15	0.63	0.80	1.88	0.81					
160	Leang Pajae	TP1	classic Maros point	chert	23.84	12.64 (broken)	3.72	0.77	2.40	4.21	0.75	2.01	3.07	1.43	1	7.18	2.25		
161	Leang Pajae	TP1	unid.	chert	14.95 (broken)	12.49	3.23	0.57	3.02	2.83	0.58		1.39	1.52					
162	Leang Pajae	TP1	classic Maros point	chert	26.27	13.97	3.63	0.87	2.86	3.97	0.61	2.26	3.82	1.74		6.87	3.08		
163	Leang Pajae	TP1	classic Maros point	chert	27.77	13.06	3.07	0.92	2.45	2.83	0.53	1.61	3.02	1.93		6.7	2.73		
164	Leang Pajae	TP1	classic Maros point	chert	23.55	15.86	3.09	0.76	2.65	3.38	0.51	2.71	3.58			6.7	4.14		
165	Leang Pajae	TP1	Pangkep	chert	27.53	18.78	5.46	2.07	1.85	4.07	0.56							heat damage	
166	Leang Pajae	TP1	Pangkep	chert	17.96	13.73	3.21	0.68	1.56	1.44									
167	Leang Pajae	TP1	Pangkep	chert	29.78	16.84	5.30	2.23	3.52	5.67									1
168	Leang Pajae	TP1	classic Maros point	chert	26.76	15.16	4.78	1.14	3.95	3.81	0.76	1.57	2.45			8.86	4.89		
169	Leang Pajae	TP1	Lompoa	chert	23.72	10.64	3.61	0.78	3.44	5.26						7.58	3.75		
170	Leang Pajae	TP1	Pangkep	chert	23.69	14.66	3.45	1.03	2.44	2.69									
171	Leang Pajae	TP1	unid.	chert	19.44 (broken)	14.11	3.29	0.70	2.85	3.40	0.72	1.57							

172	Leang Pajae	TP1	classic Maros point	chert	21.81 (broken)	11.18	3.54	0.71	3.52	5.10	0.67	0.70				8.82	2.74		
173	Leang Pajae	TP1	classic Maros point	chert	24.07	11.61	2.06	0.52	0.70	2.02	0.64					8.46	2.96		
174	Leang Pajae	TP1	classic Maros point, incomplete	chert	20.48 (broken)	18.94	3.61	1.30	3.35	4.19	0.57	1.48	2.58			7.82	3.5		
175	Leang Pajae	TP1	Pangkep	chert	26.90	16.39	5.03	1.84	2.01	1.64									
176	Leang Pajae	TP1	classic Maros point	chert	31.65	12.62	4.84	1.17	3.01	3.96	0.31	2.20	2.26			6.71	3.69	edge gloss	
177	Leang Pajae	TP1	classic Maros point	chert	22.63	17.97	4.82	1.20	3.31	3.21	1.16	0.98	3.06			9.08	2.78		
178	Leang Pajae	TP1	classic Maros point	chert	34.38	20.73	5.42	2.08	4.27	5.27	0.67	2.25	2.70			10.48	4.07		
179	Leang Pajae	TP1	Pangkep	chert	25.58	15.32	3.04	1.05	7.19	6.27									
180	Leang Pajae	TP1	classic Maros point	meta-sedamentary	18.32 (broken)	11.83	3.93	0.72	2.14	2.87	0.75	0.40				8.99	1.62		
181	Leang Pajae	TP1	classic Maros point	meta-sedamentary	25.04	14.13	4.31	1.59	2.87	5.58	0.73	1.80	4.27			6.54	2.94		
182	Leang Pajae	TP1	unid.	chert	26.58	14.01 (broken)	3.53 (broken)	0.96	2.58	2.60								heat damage	
183	Leang Pajae	TP1	Mallinrung point	chert	19.68	12.80	2.44 (broken)	0.65	1.65	3.22	0.84	1.03						heat damage	
184	Leang Pajae	TP1	unid.	chert	24.16 (broken)	13.39	3.11	1.01	3.41	3.41	0.65	1.45	3.20						
185	Leang Pajae	TP1	Pangkep	chert	25.01	16.67	3.97	1.04	2.27	3.59									
186	Leang Pajae	TP1	classic Maros point	chert	10.80	9.28	1.87	0.12	2.04	3.12	0.98	0.97	2.39			5.58	1.01		

187	Leang Pajae	TP1	classic Maros point	meta-sedamentary	22.02	10.83	3.21	0.61	2.58	3.81	0.71	1.66				7.4	3.4		
188	Leang Pajae	TP1	classic Maros point	meta-sedamentary	18.24 (broken)	15.36 (broken)	2.35	0.56	1.67	2.68	0.73	1.11	1.81			12.19	3.86		
189	Leang Pajae	TP1	unid.	chert	18.92	17.87	3.98	1.20	1.79	2.67	0.70	0.85						heat damage	
190	Leang Pajae	TP1	unid.	chert	23.89 (broken)	24.92 (broken)	3.00	1.67	4.28	3.03	1.46	2.18	2.46					heat damage	
191	Leang Pajae	TP1	unid.	chert	4.62	8.38	3.02	0.28	3.79	4.56	0.61	1.27							
192	Leang Pajae	TP1	unid.	chert	21.17	19.06	3.36	1.08	2.29	2.48	0.92	0.84							
193	Leang Pajae	TP1	unid.	chert	19.95 (broken)	9.71	3.42	0.59	2.80	3.93									
194	Leang Pajae	TP1	unid.	chert	30.77	19.27	3.52	2.05	2.43	2.58	0.68								
195	Leang Pajae	TP1	unid.	chert	17.86 (broken)	10.47	2.32	0.26	2.26	2.52	0.98	1.22							
196	Leang Pajae	TP1	unid.	chert	22.85	11.82	2.76	0.62	1.25	2.05	0.58								
197	Leang Pajae	TP1	unid.	chert	18.70 (broken)	14.20 (broken)	3.37	1.18	4.12	5.23	0.76	0.66	2.16						
198	Leang Pajae	TP1	unid.	chert	28.58	13.89	4.09	1.34	2.37	6.21	0.52	0.86	2.21						
199	Leang Pajae	TP1	unid.	chert	29.06 (broken)	16.89	3.27	1.39	3.06	4.16	0.63	1.58	3.33						
200	Leang Pajae	TP1	unid.	chert	22.02	14.29 (broken)	4.33	1.16											
201	Leang Pajae	TP1	unid.	chert	27.71 (broken)	15.40	2.83	0.79											
202	Leang Rakkoe	TP1	Lompoa	chert	28.83	19.17	4.97	2.58	8.63	14.14				not taken	not taken	9.72	1.66		1
203	Leang Rakkoe	TP1	classic Maros point	chalcedony	27.85	10.84	2.88	0.78	1.31	2.13	1.00	1.22	2.34	not taken	not taken	6.29	1.55		
204	Leang Rakkoe	TP1	classic Maros point	chert	25.08	13.40	4.25	1.17	2.35	3.68	0.97	1.50	2.55	not taken	not taken	5.82	1.9		
205	Leang Rakkoe	TP1	classic Maros point, incomplete	chert	19.14	10.33	2.93	0.51	1.05	2.80	0.68	1.03		not taken	not taken				

206	Leang Rakkoe	TP1	classic Maros point	chert	23.60	12.12	3.09	0.69	1.33	1.79	1.04			not taken	not taken				
207	Leang Rakkoe	TP1	classic Maros point, incomplete	chert	26.24	15.40	3.27	1.05	2.28	3.92	1.10	1.32		not taken	not taken				
208	Leang Rakkoe	TP1	unid.	chert	27.44	16.26	2.91	1.15	2.59	2.73	1.06	1.31		not taken	not taken				
209	Leang Rakkoe	TP1	unid.	chert	42.05 (broken)	23.32	6.51	4.54	2.26	3.25	0.80	1.02		not taken	not taken			heat damage	
210	Leang Rakkoe	TP1	unid.	chert	33.14 (broken)	8.80 (broken)	3.05	0.72	1.50	2.79	0.71	1.31		not taken	not taken				
211	Tallasa	surface find	Mallinrung point	chert	28.61	16.89	4.96	2.25	2.56	4.43	0.58	1.29	3.72	1.32				heat damage	

Table C. Dataset for osseous points.

Analysis ID	Site	Artefact No.	Material	Length (mm)	Distal tip width (mm)	Midsection width (mm)
1	Leang Bulu' Sipong 1	5253	Suidae tooth	24.77	1.39	3.49
2	Leang Bulu' Sipong 1	5528	Suidae tooth	46.02	3.57	5.12
3	Leang Bulu' Sipong 1	5	bone	11.14	3.57	4.13
4	Leang Pajae	1510	Suidae tooth	28.82	3.91	4.43
5	Leang Bulu' Sipong 1	1465	Suidae tooth	19.64	2.94	5.43
6	Leang Bulu' Sipong 1	4	Suidae tooth	15.18	0.91	3.41
7	Leang Bulu' Sipong 1	293	Suidae tooth	18.88	0.62	4.29
8	Leang Bulu' Sipong 1	974	Suidae tooth	19.15	1.89	5.18
9	Leang Pajae	3005	Suidae tooth	19.98	0.37	2.04
10	Leang Pajae	1577	Suidae tooth	16.35	0.87	1.99
11	Leang Pajae	3423	Suidae tooth	41.63	2.10	3.43
12	Leang Pajae	3316	Suidae tooth	40.95	1.20	4.78
13	Leang Pajae	1792	Suidae tooth	50.23	5.41	7.77
14	Leang Bulu' Sipong 1	6775	Suidae tooth	36.69	Obscured	3.74
15	Leang Bulu' Sipong 1	5916	Suidae tooth	36.39	1.54	4.71
16	Leang Bulu' Sipong 1	414	Suidae tooth	38.80	2.19	6.62
17	Leang Bulu' Sipong 1	3510	Suidae tooth	28.56	0.72	4.90
18	Leang Bulu' Sipong 1	--	Suidae tooth	28.04	3.02	4.58
19	Leang Bulu' Sipong 1	5833	Suidae tooth	20.64	1.86	5.02
20	Leang Bulu' Sipong 1	--	bone	20.43	1.07	3.19
21	Leang Bulu' Sipong 1	3	Suidae tooth	19.61	0.90	3.24
22	Leang Bulu' Sipong 1	--	Suidae tooth	19.33	0.83	4.46
